# Metabolic profiling stratifies colorectal cancer and reveals adenosylhomocysteinase as a therapeutic target

**DOI:** 10.1038/s42255-023-00857-0

**Published:** 2023-08-14

**Authors:** Johan Vande Voorde, Rory T. Steven, Arafath K. Najumudeen, Catriona A. Ford, Alex Dexter, Ariadna Gonzalez-Fernandez, Chelsea J. Nikula, Yuchen Xiang, Lauren Ford, Stefania Maneta Stavrakaki, Kathryn Gilroy, Lucas B. Zeiger, Kathryn Pennel, Phimmada Hatthakarnkul, Efstathios A. Elia, Ammar Nasif, Teresa Murta, Eftychios Manoli, Sam Mason, Michael Gillespie, Tamsin R. M. Lannagan, Nikola Vlahov, Rachel A. Ridgway, Colin Nixon, Alexander Raven, Megan Mills, Dimitris Athineos, Georgios Kanellos, Craig Nourse, David M. Gay, Mark Hughes, Amy Burton, Bin Yan, Katherine Sellers, Vincen Wu, Kobe De Ridder, Engy Shokry, Alejandro Huerta Uribe, William Clark, Graeme Clark, Kristina Kirschner, Bernard Thienpont, Vivian S. W. Li, Oliver D. K. Maddocks, Simon T. Barry, Richard J. A. Goodwin, James Kinross, Joanne Edwards, Mariia O. Yuneva, David Sumpton, Zoltan Takats, Andrew D. Campbell, Josephine Bunch, Owen J. Sansom

**Affiliations:** 1 Cancer Research UK Beatson Institute, Glasgow, UK; 2 National Physical Laboratory, London, UK; 3 Department of Metabolism Digestion and Reproduction, Faculty of Medicine, Imperial College London, London, UK; 4 School of Cancer Sciences, University of Glasgow, Glasgow, UK; 5 The Francis Crick Institute, London, UK; 6 Department of Human Genetics, University of Leuven, KU Leuven, Leuven, Belgium; 7 Bioscience, Early Oncology, AstraZeneca, Cambridge, UK; 8 Imaging and Data Analytics, Clinical Pharmacology and Safety Sciences, R&D, AstraZeneca, Cambridge, UK; 9 Institute of Infection, Immunity and Inflammation, College of Medical, Veterinary and Life Sciences, University of Glasgow, Glasgow, UK; 10 Biological Mass Spectrometry, Rosalind Franklin Institute, Didcot, UK; 11 Present Address: Københavns Universitet, BRIC, Copenhagen, Denmark; 12 Present Address: Rheos Medicines, Cambridge, MA USA

**Keywords:** Cancer metabolism, Experimental models of disease, Metabolism

## Abstract

The genomic landscape of colorectal cancer (CRC) is shaped by inactivating mutations in tumour suppressors such as *APC*, and oncogenic mutations such as mutant *KRAS*. Here we used genetically engineered mouse models, and multimodal mass spectrometry-based metabolomics to study the impact of common genetic drivers of CRC on the metabolic landscape of the intestine. We show that untargeted metabolic profiling can be applied to stratify intestinal tissues according to underlying genetic alterations, and use mass spectrometry imaging to identify tumour, stromal and normal adjacent tissues. By identifying ions that drive variation between normal and transformed tissues, we found dysregulation of the methionine cycle to be a hallmark of APC-deficient CRC. Loss of *Apc* in the mouse intestine was found to be sufficient to drive expression of one of its enzymes, adenosylhomocysteinase (AHCY), which was also found to be transcriptionally upregulated in human CRC. Targeting of AHCY function impaired growth of APC-deficient organoids in vitro, and prevented the characteristic hyperproliferative/crypt progenitor phenotype driven by acute deletion of *Apc* in vivo, even in the context of mutant *Kras*. Finally, pharmacological inhibition of AHCY reduced intestinal tumour burden in *Apc*^*Min/+*^ mice indicating its potential as a metabolic drug target in CRC.

## Abstract (web summary)

In this study, Vande Voorde et al. investigate the potential of untargeted metabolomics as a stratification tool for colorectal cancer (CRC). They present a comprehensive pipeline to uncover metabolic vulnerabilities in CRC based on its genetic origin. With this approach, they show perturbations in methionine metabolism linked to APC deficiency, and identify adenosylhomocysteinase as an actionable therapeutic target.

## Main

**Fig. 1:**
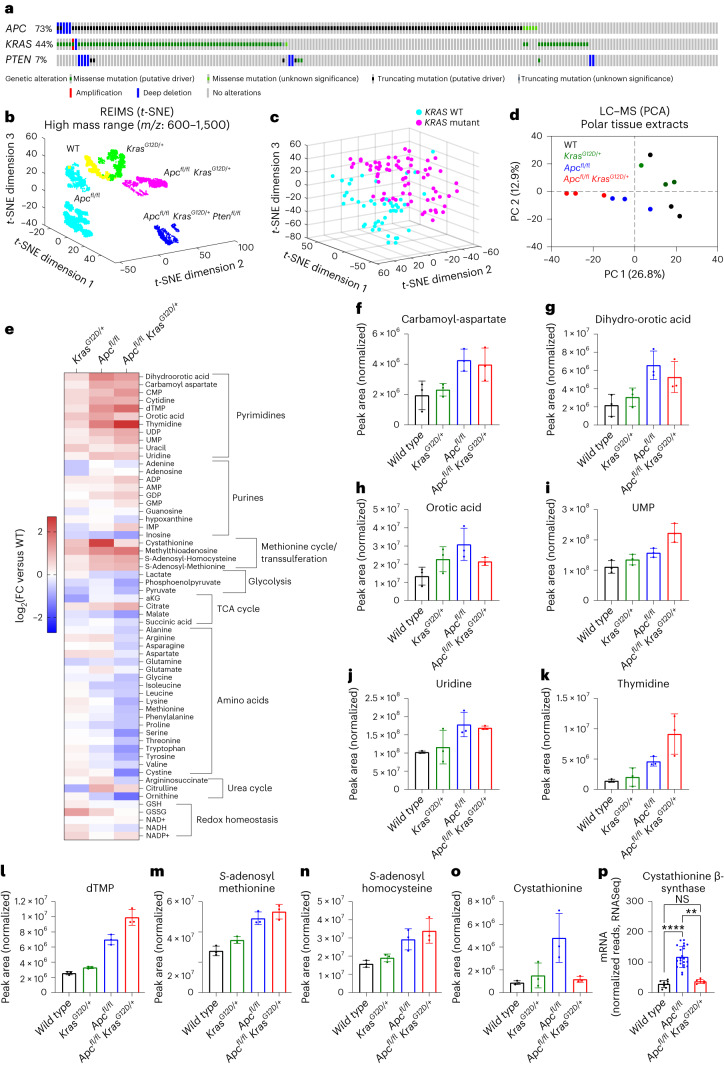
Stratification of genetically engineered mouse models of intestinal hyperproliferation by metabolic profiling. **a**, Oncoprint showing genetic alterations of *APC*, *KRAS* and *PTEN* in human colorectal adenocarcinoma (TCGA, Firehose Legacy; https://www.cbioportal.org/). **b**, *t*-SNE plot of REIMS data acquired from small intestinal epithelium after specific activation of oncogenic drivers focusing analysis on ions within a mass range of *m/z* 600–1,500. Each symbol corresponds to a single mass spectrum acquired using the REIMS forceps. Data were acquired from WT (*n* = 3), KRAS (*n* = 4), APC (*n* = 11), APC KRAS (*n* = 4) and APC KRAS PTEN (*n* = 5) mice. **c**, Three-dimensional *t*-SNE visualization of REIMS data collected from ex vivo clinical samples using only 50 significant classification features within a mass range of *m/z* 50–1,200. Each dot corresponds to a single mass spectrum described by the 50 features. Multiple spectra were collected from the same patient as technical replicates in the training data. Data were acquired from *KRAS-*WT (*n* = 8) and *KRAS*-mutant (*n* = 16) samples. **d**, PCA of untargeted LC–MS data (1,270 features) acquired from polar extracts of small intestinal tissues from WT, KRAS, APC, and APC KRAS mice (*n* = 3 for each genotype). **e**, Heat map showing differences in metabolite abundance in small intestinal tissues of KRAS, APC and APC KRAS mice compared with WT mice (targeted analysis of LC–MS data; heat map constructed based on fold change between the averages of each experimental group; *n* = 3). **f**–**o**, Plots showing normalized abundances of intermediates of de novo pyrimidine synthesis (**f**–**h**), pyrimidine nucleo(s)(t)ides (**i**–**l**) and intermediates of the methionine cycle/transsulferation pathway (**m**–**o**). In **f**–**o**, data are the mean ± s.d., and each dot represents data from an individual mouse (*n* = 3 mice for each genotype). **p**, Cystathionine β-synthase gene expression as analysed by RNA-seq in the small intestine of WT (*n* = 8), APC (*n* = 22) and APC KRAS (*n* = 6) mice. Data are the mean ± s.d., and each dot represents data from an individual mouse. Asterisks refer to *P* values obtained from Kruskal–Wallis test followed by Dunn’s correction (**P = 0.0026; ****P < 0.0001; not significant (NS), *P* > 0.9999). TCA, tricarboxylic acid. Source data

With CRC being the second most common cause of cancer-related deaths worldwide^1^, there is an urgent need for better diagnostic tools and new, more targeted therapies. Inactivation of the tumour suppressor gene adenomatous polyposis coli (*APC*) is the most common event in CRC (~70–80%), with co-occurring activation of oncogenic *KRAS* (40–50%), and/or mutations in other tumour suppressor genes (for example, *PTEN* or *TP53*) or oncogenes (for example, *PIK3CA*) being frequently observed (Fig. 1a)^2^. Previous research has shown that oncogenic events trigger metabolic reprogramming at the adenoma stage, with alterations in glycolytic intermediates, nucleotides and *S*-adenosyl-methionine (SAM) being observed^3^. Using genetically engineered mouse models and multimodal mass spectrometry-based metabolomics, we analysed the metabolic effects of frequently observed genetic alterations in CRC. Collectively, we show that the profound genotype-dependent metabolic changes may be exploited for tissue classification without need for ion identification, and we applied further data analysis to expose AHCY as a metabolic vulnerability of CRC.

**Extended Data Fig. 1:**
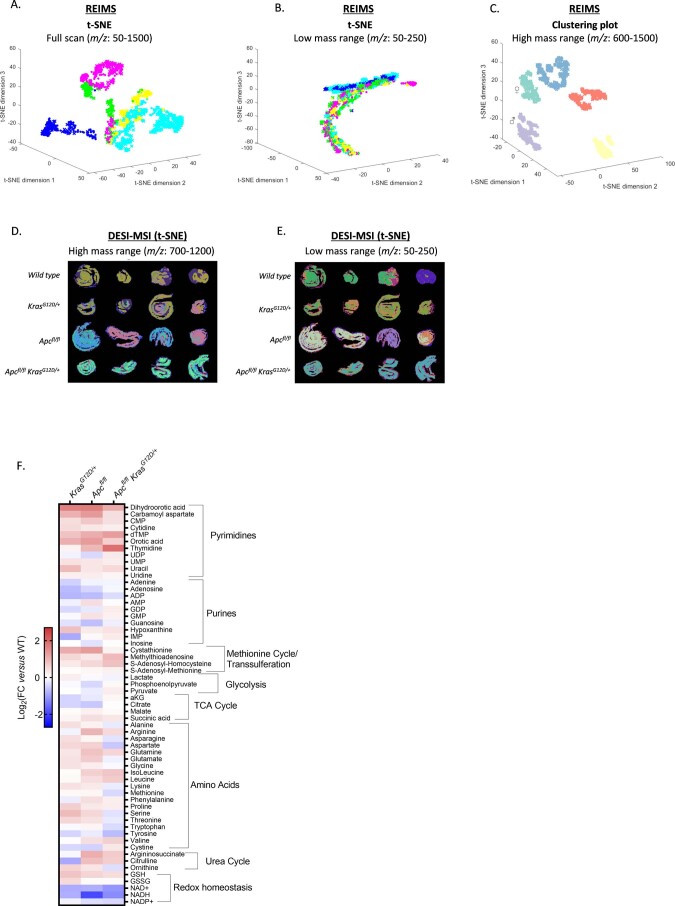
Metabolic profiling of intestinal tissues by REIMS, DESI-MSI and LC-MS. (A) Multivariate analysis of REIMS data showing segmentation when focusing analysis on the full acquired mass range (*m/z* = 50-1500), or (B) low mass range (*m/z*: 50-250). Colour coding: yellow = WT; green = KRAS; cyan = APC; pink = APC KRAS; blue = APC KRAS PTEN. Each dot corresponds to a single mass spectrum acquired using the REIMS forceps. Data acquired from WT (n = 3), KRAS (n = 4) APC (n = 11), APC KRAS (n = 4), and APC KRAS PTEN (n = 5) mice. (C) Clustering plot of REIMS data (*m/z*: 600-1500) showing genotype-dependent clustering. ♂/♀ indicate male/female dominant APC clusters. (D) t-SNE of DESI-MSI data acquired from longitudinal sections of the rolled up small intestine of WT, KRAS, APC, and APC KRAS mice focusing analysis on ions within high mass range *m/z*: 700-1200, or (E) low mass range *m/z*: 50-250 (n = 4 per genotype, each image is a rolled small intestine from one individual mouse). (F) Targeted LC-MS analysis of polar extracts of colonic tissues from KRAS, APC, and APC KRAS mice compared with WT mice. Heatmap constructed based on fold change between the averages of each experimental group (n = 3). Source data

We crossed mice expressing a tamoxifen-inducible intestine-specific Cre recombinase, under the control of the villin promoter (Villin-Cre^ERT2^), with mice harbouring various combinations of conditional alleles of inactivated *Apc* (*Apc*^*fl*^), *Pten* (*Pten*^*fl*^), and oncogenic *Kras* (*Kras*^*G12D*^). Intraperitoneal (i.p.) delivery of tamoxifen resulted in acute gene (in)activation across the intestinal epithelium, with deletion of both copies of *Apc* causing a crypt progenitor phenotype characterized by increased proliferation^4^. To study the metabolic impact of these genetic events, intestinal epithelium was extracted from wild-type (WT), Villin-Cre^ERT2^ *Kras*^*G12D/+*^ (KRAS), Villin-Cre^ERT2^*Apc*^*fl/fl*^ (APC), Villin-Cre^ERT2^ *Apc*^*fl/fl*^*Kras*^*G12D/+*^ (APC KRAS) and Villin-Cre^ERT2^ *Apc*^*fl/fl*^*Kras*^*G12D/+*^*Pten*^*fl/fl*^ (APC KRAS PTEN) mice. These were analysed by rapid evaporative ionizing mass spectrometry (REIMS), which allows rapid determination of metabolic profiles with no need for sample preparation and can therefore provide real-time tissue characterization during surgery^5^. The REIMS spectrum is dominated by abundant molecules such as phospholipids, lysolipids and fatty acids^6,7^, and, accordingly, segmentation of the REIMS data by *t*-distributed stochastic neighbour embedding (*t*-SNE) was optimal when focusing the analysis on large ions (mass range of *m/z* 600–1,500; Fig. 1b and Extended Data Fig. 1a,b). Inactivation of APC resulted in a clear metabolic differentiation from WT tissues. Additional oncogenic transformation (that is, APC KRAS and APC KRAS PTEN) resulted in distinct separation (Fig. 1b), indicating the profound impact of these clinically relevant events on the metabolic landscape of the intestine. Clustering analysis (*k*-means clustering on *t*-SNE reduced data, *k* = 5) identified genotype-dependent metabolic clusters, and sex-dependent segmentation was observed for samples derived from APC mice as indicated by a male-dominant APC cluster (containing 77% of male APC spectra) and a female-dominant APC cluster (containing 99% of female APC spectra; Extended Data Fig. 1c). In line with the lack of intestinal hyperproliferation in Kras mice when sampled at this timepoint^8^, WT and KRAS tissues co-clustered (Fig. 1b and Extended Data Fig. 1c). Tentative molecular identities were assigned to ions discriminating the various genotypes and pathway analysis showed that alterations in lipid biosynthesis were largely driving the various metabolic clusters (Supplementary Tables 1 and 2). Our data reveal a substantial effect of APC inactivation and further oncogenic transformation on the intestinal lipidome and highlight the clinical potential of using metabolic phenotyping by REIMS for intrasurgical tissue classification of CRC. To test this, we performed REIMS on samples collected from 24 individuals undergoing colorectal resection surgery (carcinomas and adenomas) for which underlying genomic alterations were determined by whole-exome sequencing. We applied a support vector machine-based algorithm to build predictive models to stratify *KRAS*-mutant (*n* = 16) and *KRAS-*WT tissues (*n* = 8). Model optimization feature refinement was effective as shown in Figure 1c, where a linear stratification of the two classes was observed with only the 50 selected features (Supplementary Table 3). The model performance was tested by exhaustive cross-validation using a leave-one-patient-out strategy, and produced accurate predictions of the *KRAS* status of individuals with true positive rate = 0.87, true negative rate = 1.00 and balanced accuracy = 0.93. Collectively, our findings add to the notion that metabolic determinants can be used for classifying subtypes of cancer^9–11^.

REIMS provides a suitable platform for rapid metabolic profiling based on abundant ions in an intact biological specimen but has predominately been used for lipidomics-based analyses^12,13^. Electrosurgical REIMS data are largely untested for detection of low-mass metabolites with relevance to cancer (for example, central carbon metabolism, amino acids and nucleotides) and, previously, poor accuracy was found when using the lower end of the mass range due to detection of a large number of fragment ions rather than intact molecules^14^. Small intestinal tissues of WT, KRAS, APC and APC KRAS mice were therefore additionally analysed using desorption electrospray ionization (DESI) mass spectrometry imaging (MSI), which can provide rich information relating to the spatial distribution of low *m/z* metabolites. As with REIMS, DESI provides a metabolic readout of intact tissues with no need for solvent-based metabolite extraction, which often selectively enriches specific classes of molecules. Similarly to what is observed with REIMS, untargeted multivariate data analysis focusing the mass range on large ions (*m/z* 700–1,200) showed distinct clustering of APC and APC KRAS, and co-clustering of WT and KRAS tissues (Extended Data Fig. 1d; n_components = 8). There were also indications of genotype-dependent clustering when restricting the analysis to smaller ions (*m/z* 50–250) suggesting additional conserved alterations in non-lipid-driven processes, which could be used for tissue classification or target identification (Extended Data Fig. 1e; n_components = 8). To investigate this further, we analysed the polar fraction of small intestinal tissue extracts by liquid chromatography–mass spectrometry (LC–MS). Unsupervised principal component analysis (PCA) showed separate clustering of APC and APC KRAS tissues, and co-clustering of WT and KRAS tissues (Fig. 1d). Targeted data analysis revealed increased intermediates of de novo pyrimidine synthesis and pyrimidine nucleo(s)(t)ides following loss of *Apc* (Fig. 1e,f–l). Furthermore, we found evidence of increased activity of the methionine cycle as indicated by increased levels of SAM, *S*-adenosyl-homocysteine (SAH) and methylthioadenosine (Fig. 1e,m,n). Similar regulation of these metabolites was also observed in colonic tissues (Extended Data Fig. 1f). These findings are in line with the previously reported upregulation of pyrimidine synthesis genes by MYC, and increased abundance of SAM in human CRC compared with paired normal tissue^3^. In addition, cystathionine levels (Fig. 1o) and gene expression of cystathionine β-synthase (*Cbs*; Fig. 1p) were increased in APC tissues, indicating carbon contribution of homocysteine to the transsulferation pathway. Previous work has shown an association between *KRAS* mutations and epigenetic silencing of *CBS* in primary CRCs^15^, and, accordingly, we found cystathionine levels and *Cbs* expression decreased in APC KRAS tissues (Fig. 1o,p).

**Fig. 2:**
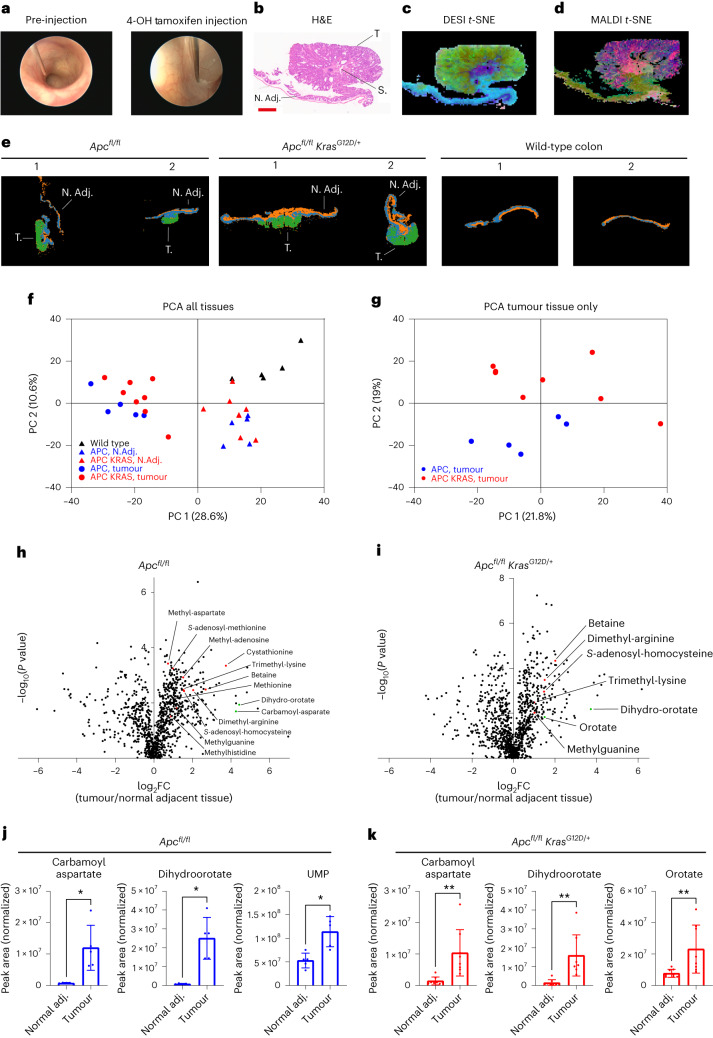
Metabolic profiling of genetically engineered mouse models of APC-deficient CRC using MSI and LC–MS. **a**, Representative images of endoscopy-guided submucosal delivery of 4-OH-tamoxifen in the mouse colon resulting in localized genetic recombination and tumour formation. **b**, Representative image of H&E-stained distal colon tissue with an *Apc*-deficient tumour. N. Adj., normal adjacent tissue; S, stroma; T, tumour tissue. Tissues analysed for *n* = 4 mice; *n* = 1 shown. Scale bar, 1 mm. **c**,**d**, *t*-SNE plot of data acquired by DESI-MSI (negative polarity) (**c**) and MALDI-MSI (negative polarity) (**d**) of distal colon tissue of locally induced APC mice (tissues analysed for *n* = 4 animals; *n* = 1 shown). **e**, *k*-means plots of data acquired by DESI-MSI (positive polarity) of distal colon tissue of locally induced APC (*n* = 5) and APC KRAS (*n* = 7) mice, and normal colon from WT (*n* = 4) mice (*n* = 2 mice per group shown). **f**, PCA of untargeted LC–MS data (1,322 features) acquired on polar extracts of normal adjacent and colon tumour tissues of APC (*n* = 5) and APC KRAS (*n* = 8) mice, and control colon of WT (*n* = 5) mice. **g**, PCA of untargeted LC–MS data acquired on polar extracts of tumour tissues of APC (*n* = 5) and APC KRAS (*n* = 8) mice. **h**,**i**, Volcano plots showing metabolic differences between paired normal adjacent and colon tumour tissues of APC (*n* = 5) or APC KRAS (*n* = 8) mice as detected by untargeted LC–MS (*P* values obtained from paired *t*-tests). All annotated metabolites: fold change ≥ 1.5 and significant after Benjamini–Hochberg false discovery rate (FDR) correction (*q* = 0.05). Red dots indicate metabolites related to methionine metabolism; green dots indicate intermediates of de novo pyrimidine biosynthesis. **j**,**k**, Plots showing normalized abundances (mean ± s.d.) of intermediates of de novo pyrimidine synthesis in tumour and normal adjacent colon tissue of APC (*n* = 5 mice) and APC KRAS (*n* = 8 mice) mice (targeted analysis of LC–MS data). Each dot represents an individual mouse. Asterisks refer to *P* values obtained from one-tailed Wilcoxon matched-pairs signed-rank tests (*P = 0.0313; **P = 0.0039). Source data

**Extended Data Fig. 2:**
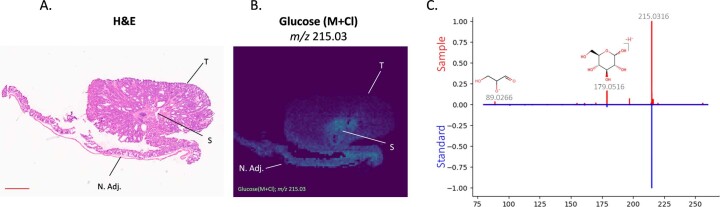
Decreased glucose abundance in tumour epithelium. (A) H&E of distal colon tissue with an *Apc*-deficient tumour [same H&E as shown in Fig. 2B and Extended Data Fig. 3A; N. Adj.: normal adjacent tissue; S: stroma; T: tumour tissue]. Tissues analysed for n = 4 animals; n = 1 shown. Scale bar: 1 mm. (B) DESI-MSI showed decreased abundance of an ion with *m/z* 215.03 in the tumour epithelial compartment of APC tumours. [N. Adj.: normal adjacent tissue; S: stroma; T: tumour tissue]. Database search (https://hmdb.ca) suggested that this could be assigned as glucose [M+Cl]^−^. A standard solution of glucose was mixed with chlorinated dopamine to obtain chlorinated glucose for comparison. (C) DESI tandem mass spectrometry showed that the fragmentation of the ion of interest produced ions matched to the fragmentation pattern of the chlorinated standard, further supporting the ID of this metabolite of interest. All highlighted *m/z* of fragments and precursor ions on the mass spectra are present in both standard and sample.

**Extended Data Fig. 3:**
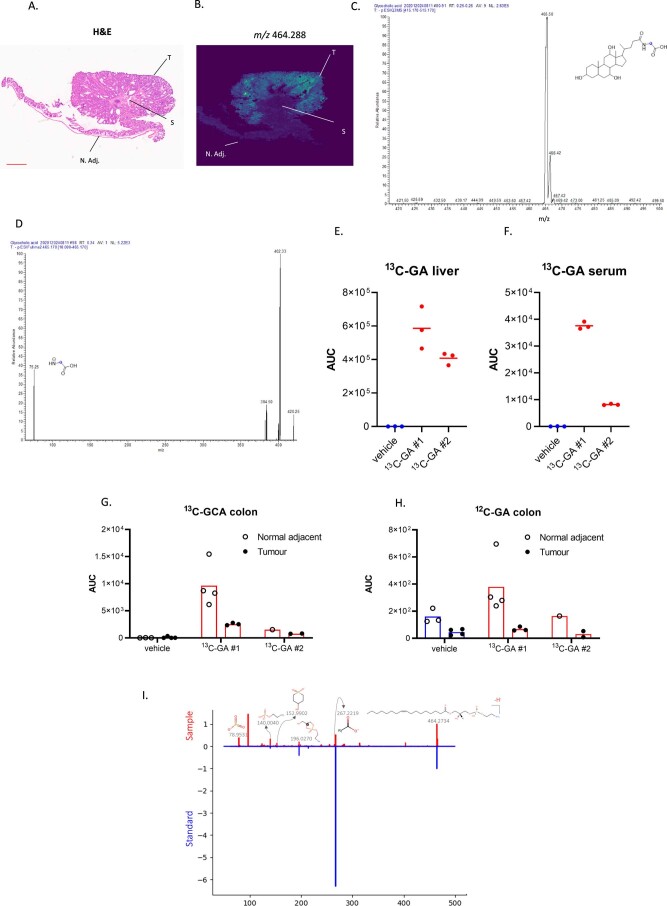
Tumour epithelial specific accumulation of Lyso-PE 17:1. (A) H&E of distal colon tissue with an *Apc*-deficient tumour [same H&E as shown in Fig. 2B and Extended Data Fig. 2A; N. Adj.: normal adjacent tissue; S: stroma; T: tumour tissue]. Tissues analysed for n = 4 animals; n = 1 shown. Scale bar: 1 mm. (B) specific accumulation of an ion with *m/z* 464.288 in the tumour epithelial compartment of APC tumours as detected by MALDI-MSI. The two top tentative parent ion identifications (that is glycocholic acid and LysoPE 17:1) obtained from a publicly available database (https://hmdb.ca) were experimentally validated. (C) A ^13^C-glycocholic acid (^13^C-GA) standard solution was infused to detect the parent ion (blue dot indicates ^13^C label) using Triple Quadrupole Mass Spectrometry, and (D) a Multiple Reaction Monitoring (MRM) method was developed to study the abundance of ^13^C-GA in tissues via its a diagnostic ion of 75 Da (blue dot indicates ^13^C label). Tumour-bearing APC mice were administered ^13^C-GA (75 mg/kg p.o.; n = 2) or vehicle (HPMC/Tween:DMSO; v-v 90:10; n = 1). After 8.5 hours, serum, liver and distal colon were harvested and processed for LC-MS. ^13^C-GA was detected in (E) liver, (F) serum, (G) normal adjacent colonic tissue and tumour tissues. No increased abundance of (G) ^13^C-GA or (H) ^12^C-GA was observed in tumour tissues compared with normal adjacent colonic tissue. Panels E-H show the mean of metabolic extractions of the multiple tissue fragments for each mouse, each dot represents data obtained from a single tissue fragment, or serum extract. Liquid extraction surface analysis tandem mass spectrometry was applied to study fragmentation of the *m/z* 464.288 precursor ion, which showed matched ions to the predicted fragmentation pattern of LysoPE 17:1, as well as to the (I) fragmentation of a commercially available standard further supporting the ID of this metabolite of interest. All highlighted *m/z* of fragments and precursor ions on the mass spectra are present in both standard and sample. Source data

These data, obtained in a short-term model of intestinal hyperproliferation, indicate specific metabolic rewiring after oncogenic transformation of the entire intestine. To test the relevance of our findings in a tumour model, we performed endoscopy-guided injection of 4-OH-tamoxifen in the colonic submucosa of APC and APC KRAS mice (Fig. 2a). This resulted in the formation of localized colonic tumours with stromal infiltration (Fig. 2b). After confirming tumour development by colonoscopy, the distal colon was dissected and analysed by both DESI-MSI and matrix-assisted laser desorption/ionization mass spectrometry imaging (MALDI-MSI) to increase metabolite coverage. Multivariate analysis segmented the different tumour compartments (Fig. 2c,d; n_components = 15 and 16, respectively), showing that MSI-based metabolic profiling can be applied to detect and discriminate tumour, stromal and normal tissue in CRC. Certain ions showed pronounced specificity for normal versus transformed tissue (Extended Data Figs. 2a,b and 3a,b). Tentative metabolite assignments were validated using a combinatorial approach of tandem mass spectrometry (MS/MS) and in vivo stable isotope tracing. This revealed specific depletion of glucose (Extended Data Fig. 2a–c) in the peripheral tumour epithelium-rich region compared with the central stromal region, or adjacent normal tissue. Conversely, we observed specific accumulation of metabolites in tumour epithelium-rich regions (for example, *m/z* 464.288 for which MS/MS data suggest lysophophatidylethanolamine (17:1) as a likely assignment; Extended Data Fig. 3a–I).

**Extended Data Fig. 4:**
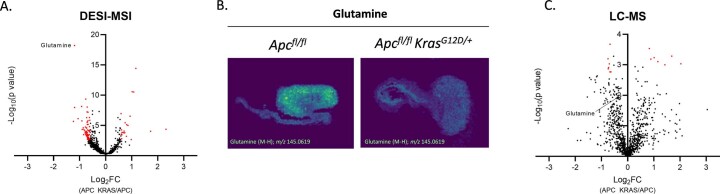
Comparative untargeted metabolomics of APC and APC KRAS tumours by DESI-MSI and LC-MS. (A) Volcano plot showing metabolic differences between tumour epithelial regions of APC (n = 2 mice) and APC KRAS (n = 2 mice) colon tumours as analysed by DESI-MSI. Red dots: FC ≥ 1.5 and significant after Benjamini-Hochberg FDR correction (q = 0.1). (B) Representative images of glutamine abundance in APC and APC KRAS colon tumours as analysed by DESI-MSI. Tissues analysed for n = 4 APC mice and n = 3 APC KRAS mice; n = 1 shown. (C) Volcano plot showing metabolic differences between bulk tumour tissue extracts of APC (n = 5 mice) and APC KRAS (n = 8 mice as analysed by untargeted LC-MS. Red dots: FC ≥ 1.5 and significant after Benjamini-Hochberg FDR correction (q = 0.1). If a more stringent FDR was used (for example q = 0.05) no metabolites pass significance for the LC/MS data. Source data

Multivariate analysis of data acquired on large colonic sections containing both tumour and normal adjacent tissue (NAT) did not distinguish between APC and APC KRAS tumours (Fig. 2e), indicating that the observed metabolic differences between tumours and NAT exceed the impact of oncogenic *Kras* expression. This was confirmed by untargeted LC–MS analysis of colon tumour tissue and paired NAT from tumour-bearing animals, and control colonic tissues from WT mice (Fig. 2f). However, restricting the PCA to tumour tissues only resulted in genotype-dependent segmentation, stratifying tumours according to *Kras* status (Fig. 2g). This indicates that careful spatial analysis of the various tumour compartments is key to understanding the metabolic impact of oncogenic drivers such as *Kras*. Therefore, we compared APC KRAS versus APC colon tumours using both DESI-MSI and LC–MS. We focused analysis of the data acquired by DESI-MSI on tumour epithelial regions only, whereas LC–MS was performed on bulk tumour tissue. Both modalities revealed specific metabolic effects of KRAS^G12D^ (Extended Data Fig. 4a,c and Supplementary Tables 4 and 5). DESI-MSI exposed a specific decrease in glutamine in APC KRAS tumour epithelium (Extended Data Fig. 4a,b), which was not revealed by untargeted LC–MS (Extended Data Fig. 4c). We recently reported the amino acid transporter SLC7A5, importing large neutral amino acids at the expense of intracellular glutamine, to be a targetable vulnerability of KRAS-mutant CRC^8^. Our current data exemplify the value of spatial metabolomics using MSI to complement bulk analysis of tumour tissue where metabolites from different compartments (that is, tumour epithelial, stromal, immune and normal adjacent cells) are mixed.

**Fig. 3:**
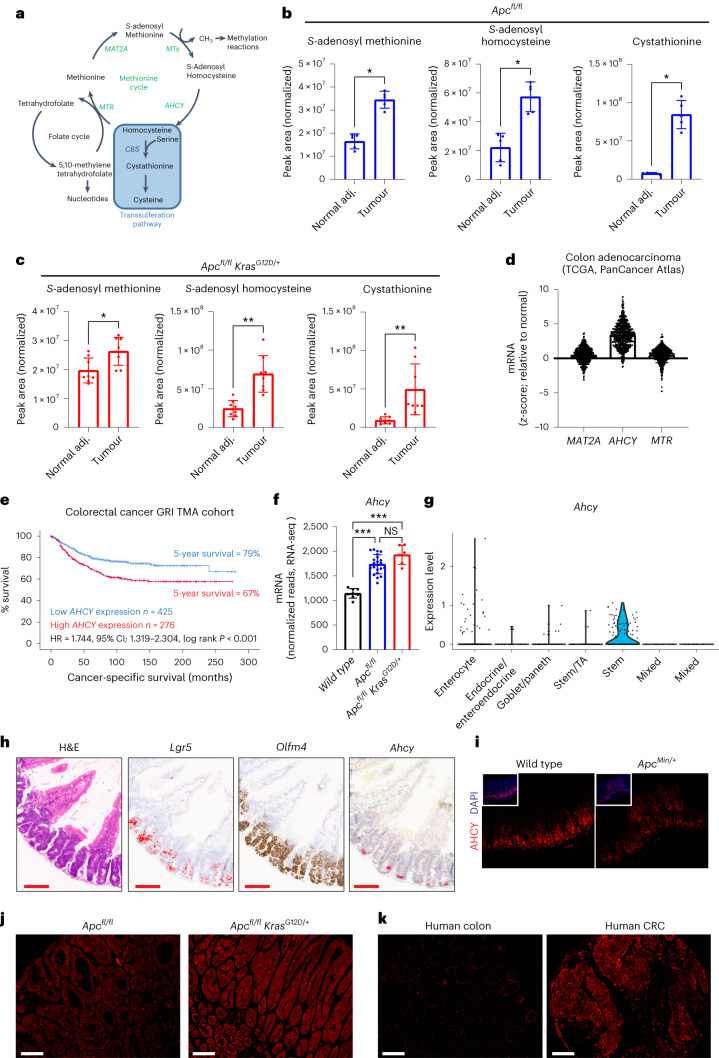
Methionine cycle activity and AHCY expression in human colorectal cancer and genetically engineered mouse models of colorectal cancer. **a**, Schematic representation of the methionine and folate cycle, and transsulferation pathway. **b**, Plots showing normalized abundances (mean ± s.d.) of SAM, SAH and cystathionine in tumour and normal adjacent colon tissue of APC mice (*n* = 5) as detected by targeted analysis of LC–MS data. Each dot represents an individual mouse. Asterisks refer to *P* values obtained from one-tailed Wilcoxon matched-pairs signed-rank tests (*P = 0.0313). **c**, Plots showing normalized abundances (mean ± s.d.) of SAM, SAH and cystathionine in tumour and normal adjacent colon tissue of APC KRAS (*n* = 8) mice as detected by targeted analysis of LC–MS data. Each dot represents an individual mouse. Asterisks refer to *P* values obtained from one-tailed Wilcoxon matched-pairs signed-rank tests (*P = 0.0273; **P = 0.0039). **d**, Expression of genes encoding enzymes of the methionine cycle in human colorectal adenocarcinoma (*n* = 592 individuals) compared with normal colon (TCGA, PanCancer Atlas; https://www.cbioportal.org/). Each dot shows the expression level for an individual. **e**, Cancer-specific survival analysis in the context of tumour epithelial *AHCY* expression in individuals with CRC (GRI TMA cohort, *n* = 701). **f**, *Ahcy* expression (mean ± s.d.) in the small intestine of WT (*n* = 8), APC (*n* = 22) and APC KRAS (*n* = 6) mice. Each dot represents an individual mouse. Asterisks represent *P* values obtained from Kruskal–Wallis test followed by Dunn’s correction (***P < 0.001: WT versus APC, *P* = 0.0004; WT versus APC KRAS, *P* = 0.0001; APC versus APC KRAS, *P* = 0.5054). **g**, *Ahcy* expression across the different cell populations of the small intestinal epithelium in APC mice (*n* = 2), as determined by scRNA-seq. **h**, H&E-stained and representative images of ISH for *Olfm4*, *Lgr5* and *Ahcy* in the small intestine of APC mice. (Tissues analysed for *n* = 4; *n* = 1 shown). Scale bar, 100 µm. Images for *Lgr5* were processed using ImageJ to show ISH staining in red. **i**–**k**, IF showing AHCY protein expression in the small intestine of WT and *Apc*^*Min/+*^ mice (tissue analysed for *n* = 2 WT and *n* = 3 *Apc*^*Min/+*^ mice; **i**), APC and APC KRAS tumours (tissues analysed for *n* = 3 animals; *n* = 1 shown; **j**), and normal human colon and human CRC (image derived from single patient sample, from a set of 49 samples analysed; **k**). Scale bars, 100 µm. Source data

**Extended Data Fig. 5:**
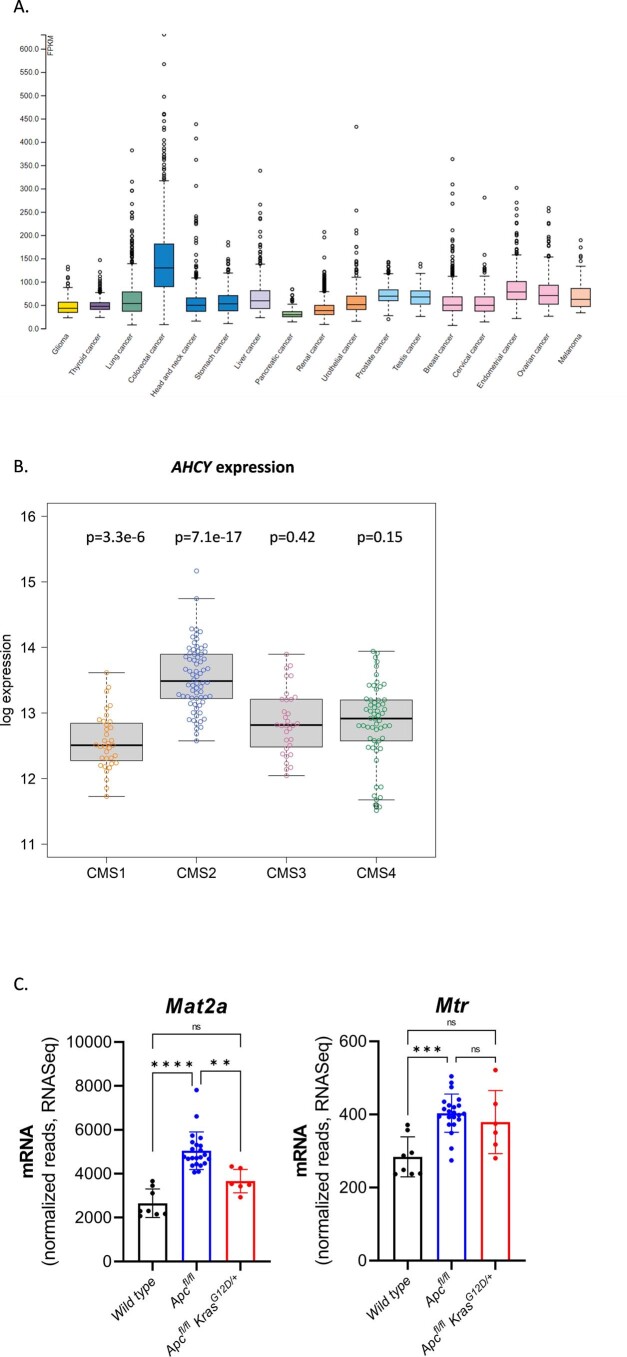
Expression of methionine cycle enzymes in human cancers and CRC GEMMs. (A) Pan-cancer analysis showing *AHCY* gene expression in 17 cancers as analysed by RNA-Seq. Data reported as FPKM (number fragments per kilobase of exon per million reads), generated by The Cancer Genome Atlas (TCGA). Image credit: Human Protein Atlas, image available from v22.proteinatlas.org^18^. Sample numbers for each cancer type are available at URL: https://www.proteinatlas.org/ENSG00000101444-AHCY/pathology Box plots show median, and 25^th^ and 75^th^ percentiles. Points are displayed as outliers if they are above or below 1.5 times the interquartile range. Minima, Maxima and n number are shown in ‘Source Data’. (B) Expression of *AHCY* across the different consensus molecular subtypes of human CRC^19^. Box plots show median, and 25^th^ and 75^th^ percentiles. Whiskers for boxplots are 1.5x the interquartile range. p-values refer to difference between each CMS group relative to all others. Two-sided p-values were calculated by fitting a linear model, computing moderated t-statistics and then correcting for multiple testing using the Benjamini-Hochberg method. Minima, Maxima and n number are shown in ‘Source Data’. (C) *Mat2a* and *Mtr* expression (Mean ± SD) in the small intestine of WT (n = 8), APC (n = 22) and APC KRAS (n = 6) mice. Each dot represents an individual mouse, asterisks represent p-values obtained from Kruskal-Wallis test followed by Dunn’s correction [(*Mat2a:* WT vs APC: p < 0.0001; WT vs APC KRAS: p = 0.9685; APC vs APC KRAS: p = 0.0095), (*Mtr*: WT vs APC: p = 0.0005; WT vs APC KRAS: p = 0.1048; APC vs APC KRAS: p > 0.9999)]. Source data

**Extended Data Fig. 6:**
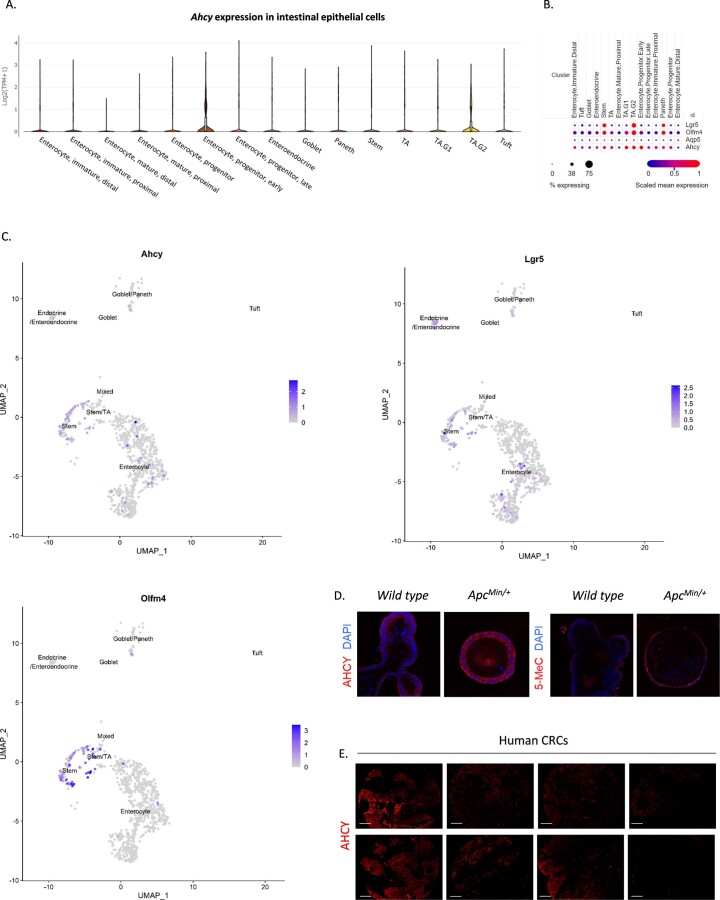
Expression of *Ahcy* and established stem cell markers in the wild type and APC-deficient murine intestine, and AHCY protein expression. (A) Violin plot showing *Ahcy* gene expression across the different cell populations of the wild type mouse small intestine^20^ (Image available from: https://singlecell.broadinstitute.org/single_cell/study/SCP44/small-intestinal-epithelium?genes=Ahcy&tab=distribution#study-visualize). (B) Dot plot showing *Lgr5, Olfm4, Ahcy*, and *Aqp5* expression across the different cell populations of the wild type mouse small intestine^20^ (Image available from: https://singlecell.broadinstitute.org/single_cell/study/SCP44/small-intestinal-epithelium?genes=lgr5%2Colfm4%2Caqp5%2CAhcy&tab=distribution#study-visualize). (C) UMAP plots showing expression of *Ahcy*, *Lgr5*, and *Olfm4* across the different cell populations of the *Apc-*deficient mouse small intestine. (data from n = 2 APC mice) (D) IF showing AHCY protein expression and abundance of 5-methylcytosine in organoids derived from the intestine of WT mice, and from adenomas in *Apc*^*Min/+*^ mice. Image from a single experiment with 4 technical replicates. (E) IF showing AHCY expression in human CRC. Scale bars: 100 µm. (Images derived from 8 CRC samples, from a cohort of 49 CRC samples analysed).

We next focused on tumour-specific polar metabolic alterations by analysing paired tumour and normal adjacent colonic tissues. This revealed increased levels of dihydroorotate (Fig. 2h,i), and other intermediates of de novo pyrimidine synthesis in APC and APC KRAS tumours (Fig. 2j,k). Also, there was a striking tumour-specific enrichment in methionine-related metabolites, and methylated metabolites requiring SAM (Fig. 2h,i). These data confirmed what we observed in the acute model of intestinal hyperproliferation (Fig. 1e–o). Given the well-described effects of interfering with pyrimidine biosynthesis and salvage in CRC (for example, by antimetabolites), we focused our attention on the methionine cycle (Fig. 3a). Targeted data analysis showed that SAM and SAH levels were significantly increased in tumours compared to paired NAT of both APC (Fig. 3b) and APC KRAS mice (Fig. 3c). Also, there was a tumour-specific increase in cystathionine (Fig. 3b,c). We analysed publicly available transcriptomic data from The Cancer Genome Atlas (TCGA; PanCancer Atlas) to understand which enzymes of the methionine cycle are transcriptionally regulated in human CRC, and found increased *AHCY* (adenosylhomocysteinase or SAH hydrolase) expression in colorectal adenocarcinoma compared to normal colon tissue (Fig. 3d). AHCY converts SAH into homocysteine, and its expression is regulated by MYC^16^. AHCY activity is required for MYC-induced mRNA Cap methylation and protein synthesis^16^, and its gene expression correlates with DNA methylation state in tumours^17^. Pan-cancer analysis across 17 different cancer types showed the highest expression of *AHCY* in CRC (Extended Data Fig. 5a)^18^. We analysed samples from a retrospective cohort of individuals with stage I–III CRC for tumour epithelial *AHCY* expression, and found that high expression was correlated with reduced cancer-specific survival (Fig. 3e). Molecular profiling of human CRC revealed enrichment of *AHCY* expression in consensus molecular subtype (CMS) 2 (Extended Data Fig. 5b), which is characterized by WNT and MYC activation, and accounts for 37% of CRCs^19^. We found acute loss of *Apc* to be sufficient to drive *Ahcy* overexpression in the mouse small intestine (Fig. 3f). Increased expression of *Ahcy* compared to WT tissues was maintained following activation of oncogenic KRAS (Fig. 3f), but not for methionine adenosyltransferase 2A (*Mat2a*) and methionine synthase (*Mtr*; Extended Data Fig. 5c). Publicly available single-cell RNA-sequencing (scRNA-seq) data of WT mouse small intestinal epithelium^20^ showed *Ahcy* expression to be enriched in cell populations that make up the intestinal crypt (Extended Data Fig. 6a,b), and we performed scRNA-seq of small intestinal epithelium from APC mice, which demonstrated predominant expression in stem cells (Fig. 3g and Extended Data Fig. 6c). Analysis of the small intestine of APC mice using in situ hybridization (ISH) confirmed *Ahcy* expression in the expanded crypt area, which is characterized by stem cell markers such as *Lgr5* and *Olfm4* (Fig. 3h). In agreement with the above, AHCY protein expression was enriched in the intestinal crypt area of WT animals as shown by immunofluorescence (IF; Fig. 3I). Furthermore, high AHCY expression was also observed in adenomas of *Apc*^*Min/+*^ mice (Fig. 3I), and in *Apc*-deficient compared with WT organoids (Extended Data Fig. 6d). Notably, IF also showed increased levels of 5-methylcytosine in *Apc*-deficient organoids, which requires SAM as a methyl donor and therefore methionine cycle activity (Extended Data Fig. 6d). Finally, tumour epithelial AHCY expression was confirmed in APC and APC KRAS tumours (Fig. 3j) and in human colon and CRC (Fig. 3k), with variation in expression between samples (Extended Data Fig. 6e).

**Fig. 4:**
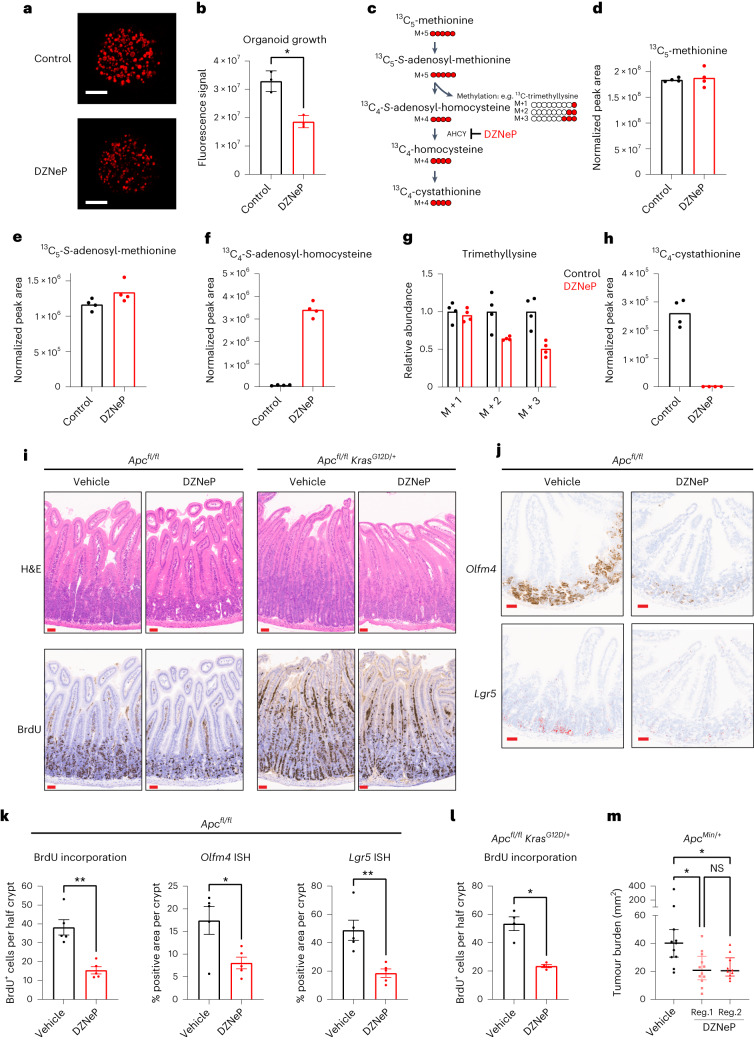
Inhibition of AHCY suppresses proliferation, stem cell expansion and tumorigenesis in APC-driven models of colorectal cancer. **a**,**b**, Representative images (scale bar, 250 µm; **a**) and quantification (**b**) of APC organoids (±DZNeP 1 μM) stained with Syto 60 nucleic acid stain (mean ± s.d.; each dot represents the mean of three independent experiments with 4 or 5 technical replicates each). Asterisk refers to *P* value obtained from one-tailed Mann–Whitney test (*P = 0.05). **c**, Schematic showing carbon contribution of ^13^C_5_-methionine to intermediates of the methionine cycle, cystathionine and trimethyllysine. **d**–**h**, Abundance of ^13^C_5_-methionine (**d**), ^13^C_5_-SAM (**e**), ^13^C_4_-SAH (**f**), various isotopologues of trimethyllysine (**g**) and ^13^C_4_-cystathionine (**h**) in APC organoids (±DZNeP 1 μM; bar indicates the mean; data from a representative experiment performed twice, with four technical replicates each; each dot represents a technical replicate). **i**, Representative images of H&E staining and IHC for BrdU on small intestinal sections of APC (*n* = 5) and APC KRAS (*n* = 4) mice treated with vehicle or DZNeP (5 mg per kg body weight). Scale bars, 50 μm; **j**, Representative images of ISH for *Olfm4* and *Lgr5* expression in the small intestine of APC mice (*n* = 5) treated with vehicle or DZNeP (5 mg per kg body weight). Scale bars, 50 μm. Images for *Lgr5* were processed using ImageJ to show ISH staining in red. **k**,**l**, Quantification of IHC for BrdU in the small intestine of APC and APC KRAS mice treated with vehicle or DZNeP (5 mg per kg body weight). Mean ± s.e.m.; *n* = 5 (APC) or *n* = 4 (APC KRAS) mice per experimental arm; each dot represents the average number of BrdU-positive cells per half crypt for each mouse), and ISH for *Olfm4* and *Lgr5* in APC mice treated with vehicle or DZNeP (5 mg per kg body weight). Mean ± s.e.m., *n* = 5 APC mice per experimental arm; each dot represents the average percentage of positive area *Olfm4* or *Lgr5* per crypt for each mouse. Asterisks refer to *P* values obtained from one-tailed Mann–Whitney tests: **k**, *P = 0.0476; **P = 0.0040; **l**, *P = 0.0143. **m**, Small intestinal macroscopic tumour burden in *Apc*^*Min/+*^ mice treated with vehicle or DZNeP from day 50 until day 85 of age. Vehicle (*n* = 11): i.p. PBS. Regime 1: DZNeP (2 mg per kg body weight i.p.; *n* = 12) using weekly cycles of 4 d of daily treatment followed by 3 d of no treatment. Regime 2: DZNeP (5 mg per kg body weight i.p.; *n* = 10) twice per week. Plot shows the median with interquartile range. Each dot represents an individual mouse. Asterisks represent *P* values obtained from Kruskal–Wallis test followed by Dunn’s correction (*P < 0.05; vehicle versus reg.1: *P* = 0.0114; vehicle versus reg.2: *P* = 0.0243; reg.1 versus reg.2: *P* > 0.999). Source data

**Extended Data Fig. 7:**
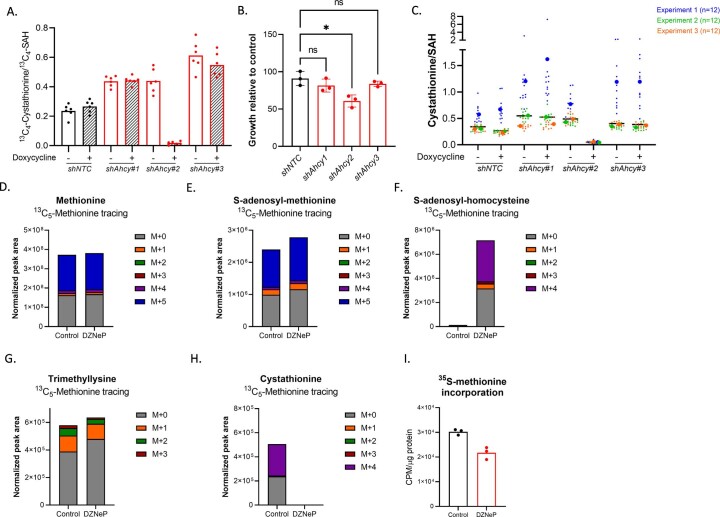
Genetic silencing of *Ahcy*, ^13^C-methionine tracing and ^35^S-protein incorporation in APC organoids. (A) Knockdown validation assay (seeding density 5,000 cells) showing that genetic silencing of *Ahcy in* APC organoids was efficient for sh*Ahcy#2* as indicated by a reduced ratio of ^13^C_4_-Cystathionine/^13^C_4_-SAH. Bar indicates Mean. Data from a single experiment. Each dot represents a technical replicate (n = 6 per condition). (B) Quantification of APC organoid growth showing growth inhibition for sh*Ahcy#2* (seeding density 2,000 cells, growth quantified with Syto 60 Nucleic Acid Stain and represented as relative to doxycycline-free culture condition). Mean ± SD; each dot represents the mean of 3 independent experiments with 12 technical replicates per condition; asterisk refers to p-value obtained from Kruskal-Wallis test followed by Dunn’s correction (*shNTC vs shAhcy1*: p = 0.9245; *shNTC vs shAhcy2*: p = 0.0276; *shNTC vs shAhcy3*: p > 0.9999). (C) Metabolomics accompanying growth assays represented in panel B showing efficient *Ahcy* knockdown for sh*Ahcy#2* as indicated by a reduced ratio of Cystathionine/SAH. Data shown for 3 independent experiments. Small dots represent technical replicates (n = 12 per condition). Large dots represent the averages for each independent experiment. (D-H) Plots accompanying Fig. 4(D-H) showing the abundance of all isotopologues for (D) methionine, (E) SAM, (F) SAH, (G) trimethyllysine, and (H) cystathionine in APC organoids (+/- DZNeP 1 μM) cultured in the presence of ^13^C_5_-methionine. Data from a representative experiment performed twice, with 4 technical replicates each. Bars show mean. (I) ^35^S-methionine protein incorporation assay performed with APC organoids (+/- DZNeP 1 μM). Data from a representative experiment performed twice, with 3 technical replicates each (shown). Bars show mean. Source data

The relevance of methionine cycle enzymes has been studied in the context of different cancers. MAT2A is a synthetic lethal target in cancers with genomic deletion of methylthioadenosine phosphorylase (*MTAP*)^21–24^, and was shown to be a metabolic vulnerability of tumour-initiating cells in lung cancer^21–25^. The methionine cycle also supports the regeneration of tetrahydrofolate via methionine synthase, which was recently found essential to support nucleotide synthesis and tumour cell proliferation under physiological levels of folate^26,27^. In addition, methionine restriction sensitizes to chemotherapy and radiation by disrupting one-carbon metabolism^28^. Our data indicate functional activity of the methionine cycle in APC-mutant CRC, and that AHCY may be important for its regulation. To test the effect of pharmacological AHCY inhibition, crypt cells were isolated from the small intestine of APC mice and cultured in vitro as organoids. Treatment with the AHCY inhibitor 3-deazaneplanocin A (DZNeP)^29–31^ or genetic silencing of *Ahcy* using lentiviral inducible shRNA significantly impaired organoid growth (Fig. 4a,b and Extended Data Fig. 7a–c). To understand the metabolic effects of AHCY inhibition in these organoids, we traced the fate of ^13^C_5_-methionine in the presence/absence of DZNeP (Fig. 4c). Whereas the intracellular levels of methionine or SAM were not affected (Fig. 4d,e and Extended Data Fig. 7d,e), we observed a pronounced increase in SAH, the substrate of AHCY, after treatment (Fig. 4f and Extended Data Fig. 7f). DZNeP decreased labelling of trimethyllysine from methionine (Fig. 4g and Extended Data Fig. 7g). This demonstrates that AHCY inhibition reduces methyltransferase activity, which may affect DNA/RNA, protein and metabolite methylation. We could not detect homocysteine in these samples but found the intracellular levels of cystathionine to be markedly reduced following treatment with DZNeP (Fig. 4h and Extended Data Fig. 7h) indicating a decreased contribution of methionine-derived carbons to the transsulferation pathway. Also, and in line with previous observations^16^, DZNeP reduced ^35^S-methionine incorporation into proteins indicating decreased protein synthesis capacity (Extended Data Fig. 7I).

**Extended Data Fig. 8:**
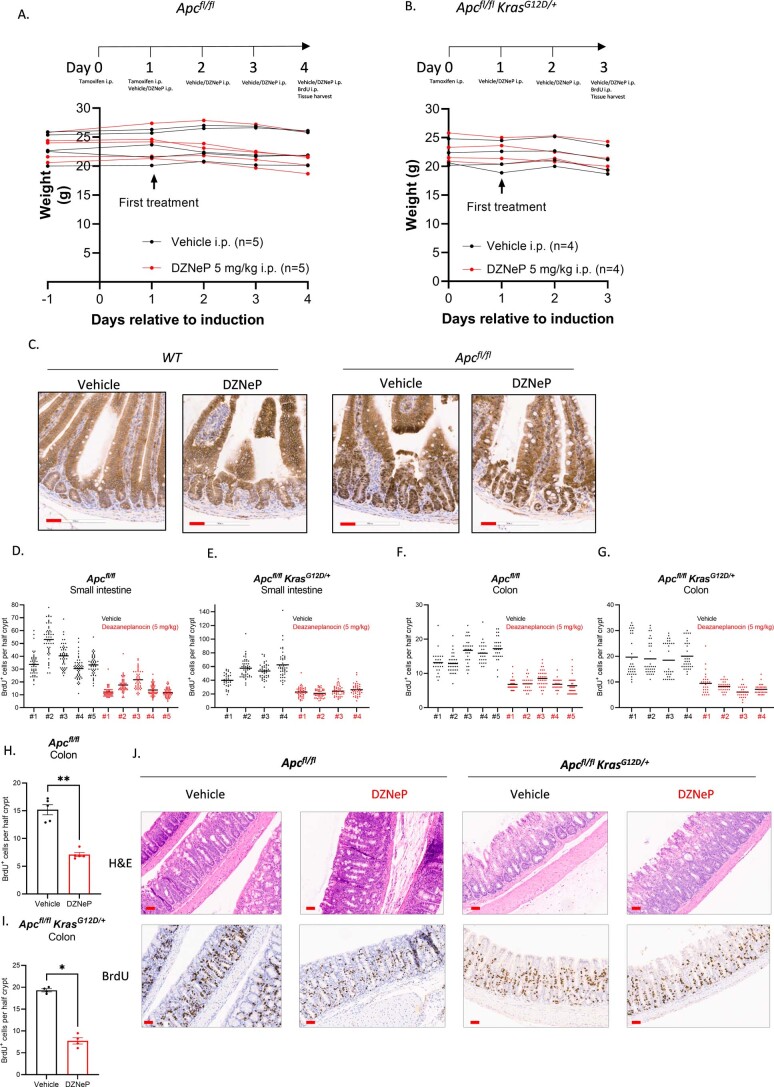
Effects of DZNEP on β-catenin localization and intestinal proliferation in APC and APC KRAS mice. (A,B) Schematic representation of experiments performed to test the effect of DZNeP in APC and APC KRAS mice, and the effect of daily treatment for 4 days or 3 days for APC and APC KRAS mice, respectively on body weight. (C) Representative images of IHC for β-catenin in the small intestine of WT (n = 3) and APC (n = 5) animals treated with vehicle or DZNeP (5 mg/kg). Scale bars (red): 50 μm. (D-G) Quantification of IHC for BrdU incorporation in the (D,E) small intestine and (F,G) colon of APC and APC KRAS mice treated with vehicle or DZNeP (5 mg/kg). [n = 5 (APC) or n = 4 (APC KRAS) mice per experimental arm]; Individual data shown for each mouse. Line represents mean. Each dot represents the number of BrdU positive cells counted per half crypt. (H,I) Quantification of IHC for BrdU in the colon of APC and APC KRAS mice treated with vehicle or DZNeP (5 mg/kg). [n = 5 (APC) or n = 4 (APC KRAS) mice per experimental arm]; Mean ± SEM. Each dot represents the average number of BrdU positive cells per half crypt for each mouse. Asterisks refer to p-values obtained from 1-tailed unpaired Mann-Whitney tests (*: p = 0.0143; **: p = 0.0040). (J) Representative images of H&E staining and IHC for BrdU on colon sections of APC (n = 5) and APC KRAS (n = 4) mice treated with vehicle or DZNeP (5 mg/kg). Scale bars: 50 μm. Source data

**Extended Data Fig. 9:**
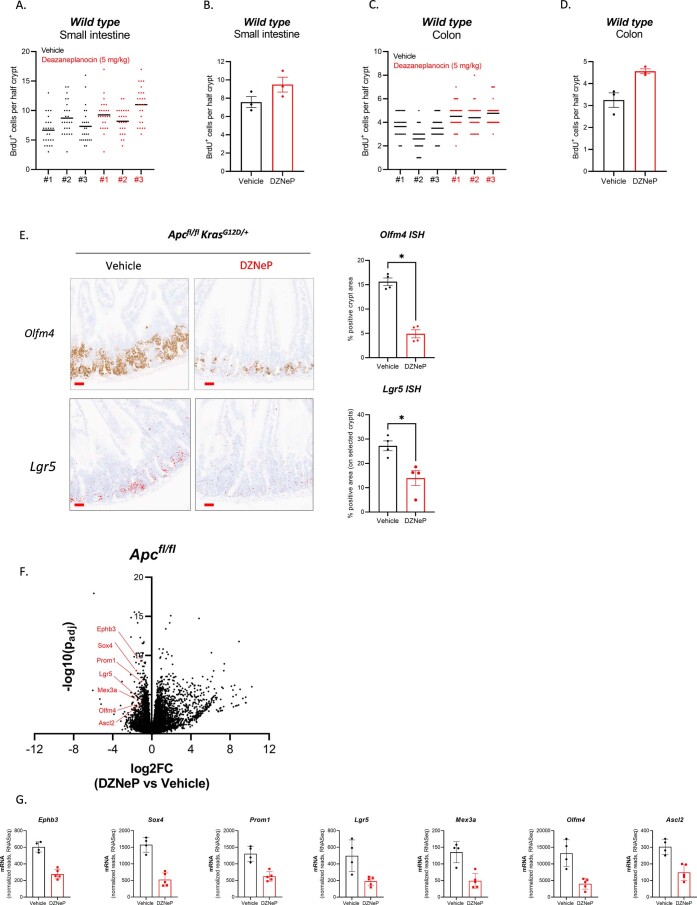
Effect of DZNeP on intestinal proliferation in WT mice and on stem cell markers in APC and APC KRAS mice. Quantification of IHC for BrdU incorporation in the (A,B) small intestine and (C,D) colon of WT mice treated with vehicle or DZNeP (5 mg/kg). (n = 3 mice per experimental arm; panels A,C: each dot represents the number of BrdU positive cells counted per half crypt. Line represents mean; panels B,D: Mean ± SEM, each dot represents the average number of BrdU positive cells per mouse. (E) Representative images and quantification of ISH for *Olfm4* and *Lgr5* expression in the small intestine of APC KRAS mice (n = 4) treated with vehicle or DZNeP (5 mg/kg). Scale bars: 50 μm. [Mean ± SEM). Each dot represents the average % positive area per crypt for each mouse. Asterisks refer to p-values obtained from 1-tailed Mann-Whitney tests (*: p = 0.0143). Images for *Lgr5* were processed using imageJ to show ISH staining in red. (F) Volcano plot showing transcriptomic changes in small intestinal tissues of APC mice treated with vehicle (n = 4) or DZNeP (5 mg/kg; n = 5) for 4 days. (G) Plots showing changes in expression of stem cell markers in small intestinal tissues of APC mice treated with vehicle (n = 4) or DZNeP (5 mg/kg; n = 5) for 4 days. (Mean ± SD). Each dot represents values obtained for an individual mouse. Source data

To study the effect of AHCY inhibition in vivo, APC and APC KRAS mice were treated with DZNeP (5 mg per kg body weight) after i.p. induction with tamoxifen (Extended Data Fig. 8a,b). The APC protein is a component of the β-catenin destruction complex and plays a critical role in maintenance of the stem cell niche and tumour suppression in the intestinal epithelium. APC negatively regulates WNT/TCF signalling by directing the degradation of β-catenin^32^. *APC* deficiency results in accumulation of nuclear β-catenin and thereby increasing transcription of its target genes (including *MYC* and *AXIN2*)^33,34^. Previously, we have shown that acute loss of *Apc* leads to a crypt progenitor phenotype with increased proliferation, stem cell markers and perturbed differentiation^35,36^. DZNeP did not affect direct WNT pathway activation as analysed by immunohistochemistry (IHC) analysis of nuclear accumulation of β-catenin in APC mice (Extended Data Fig. 8c) but significantly suppressed intestinal hyperproliferation in both the small intestine (Fig. 4i,k,l and Extended Data Fig. 8d,e) and colon (Extended Data Fig. 8f–j), without impairing intestinal crypt proliferation in WT animals (Extended Data Fig. 9a–d). Given the high expression of AHCY in intestinal crypts, we analysed the expression of intestinal stem cell markers using ISH in APC and APC KRAS mice. Expression of both *Olfm4* and *Lgr5* was significantly reduced upon DZNeP treatment (Fig. 4j,k and Extended Data Fig. 9e). RNA-seq confirmed decreased expression of additional established stem cell markers with relevance to cancer in DZNeP-treated APC mice (Extended Data Fig. 9f,g). Taken together, these findings show that AHCY inhibition prevents the crypt progenitor phenotype driven by *Apc* loss, both in the presence and absence of *Kras*^*G12D*^ expression.

**Extended Data Fig. 10:**
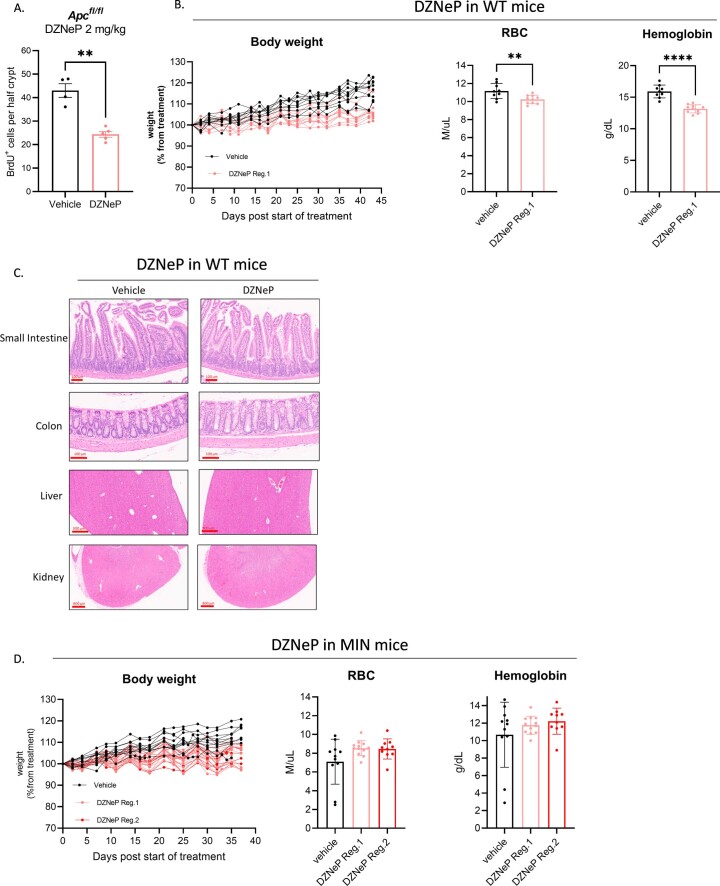
Evaluation of long-term DZNeP treatment in WT and MIN mice. (A) Quantification of IHC for BrdU in the small intestine of APC mice treated with vehicle (n = 4) or DZNeP (2 mg/kg; n = 5) for 4 days (Mean ± SEM). Each dot represents the average number of BrdU positive cells per half crypt for each mouse. Asterisk refers to p-value obtained from 1-tailed Mann-Whitney test (**: p = 0.0079). (B-C) Body weight trajectories, blood analysis (Mean ± SD) and representative images of organs from WT mice treated with vehicle or DZNeP from day 50 until day 93 of age. Data show that body weights of DZNeP-treated mice were stable, although lower than vehicle-treated animals at the end of the experiment. Blood profiling showed a decrease in red blood cells and haemoglobin in DZNeP-treated mice, but no intestinal bleeding or clinical signs of anaemia were observed. Histological analysis of small intestine, colon, liver and kidneys did not reveal any abnormalities associated with DZNeP treatment. Vehicle (n = 10 for body weight; n = 9 for blood analysis): i.p. PBS. DZNeP Regime 1 (n = 10): 2 mg/kg i.p. using weekly cycles of 4 days of daily treatment followed by 3 days of no treatment. Each dot represents an individual mouse, asterisks represent p-values obtained from 1-tailed Mann-Whitney test (**: p = 0.0076; ****: p < 0.0001). (D) Body weight trajectories and blood analysis (Mean ± SD) from *Apc*^*Min/+*^ mice treated with vehicle or DZNeP from day 50 until day 85 of age. Body weights remained stable during treatment, and DZNeP did not result in more severe disease-related anaemia. Vehicle (n = 11): i.p. PBS. Regime 1: DZNeP (2 mg/kg i.p.; n = 12) using weekly cycles of 4 days of daily treatment followed by 3 days of no treatment. Regime 2: DZNeP (5 mg/kg i.p.; n = 10) twice per week. Each dot represents an individual mouse. Source data

Previously, dietary restriction of methyl donors has been shown to reduce tumour burden in *Apc*^*Min/+*^ mice^37^. We next tested whether AHCY inhibition could reduce intestinal tumorigenesis in this model. A lower dose of DZNeP (2 mg per kg body weight) was found to be therapeutic in APC mice (Extended Data Fig. 10a), and well tolerated in WT mice for long-term treatment (Extended Data Fig. 10b,c). We next applied two distinct DZNeP treatment regimes in *Apc*^*Min/+*^ mice which significantly reduced tumour burden compared to vehicle-treated animals (Fig. 4m and Extended Data Fig. 10d). Collectively, these data show that AHCY inhibition truncates the methionine cycle and thereby reduces proliferation and tumorigenesis of APC-deficient cells indicating its potential as an actionable target for APC-driven CRC, even in the context of mutant KRAS.

In conclusion, we applied multimodal mass spectrometry-based metabolomics to investigate the metabolic consequences of common oncogenic events in CRC. We show that various combinations of genetic alterations in *Apc*, *Kras* and *Pten* perturb intestinal epithelial metabolism to the extent that metabolic profiling can accurately stratify tissues according to underlying genetic events, with no need for further ion identification. We used REIMS as a tool for rapid lipidomic segmentation highlighting the clinical potential of our results, and applied MSI to classify tumour epithelial, tumour stromal and NAT cells. Finally, untargeted LC–MS indicated increased activity of the methionine cycle in APC-mutant CRC, which ultimately revealed AHCY as a promising target for CRC. Our results argue for using a combination of these various analytical platforms to study metabolic rewiring in cancer.

## Methods

### Mouse studies

All in vivo experiments were carried out in accordance with the UK Home Office regulations (under project licences 70/8646, and PP3908577 and P609116C5) with approval from the Animal Welfare and Ethical Review Board of the University of Glasgow and the Francis Crick Institute. Mice were housed under a 12-h light–dark cycle, at constant temperature (19–23 °C) and humidity (55% ± 10%). Standard diet and water were available ad libitum. Mice were euthanized humanely either at a predefined time point preceding signs of discomfort/tumour development, or when displaying clinical signs characteristic of intestinal tumour burden as defined in the relevant licensing documents. Severity limits were adhered to at all times. The majority of the work was performed in the C57BL/6J background. The following alleles were used in this study: Villin-*CreER*^38^, *Apc*^*fl*^(ref. ^39^), *Kras*^*G12D*^(ref. ^40^), *Pten*^*fl*^(ref. ^41^) and *Apc*^*Min*^(ref. ^42^). Supplementary Table 6 summarizes the sample sizes for in vivo experiments. For conditional alleles, robust recombination throughout the intestinal epithelium was obtained by one or two i.p. injections of 2 mg tamoxifen, and tissues were collected 3 or 4 d after induction. To drive spatially localized Cre induction in the colon, 4-hydroxytamoxifen (70 μl, 100 nM; Merck Millipore) was delivered under general anaesthesia via a single injection into the colonic submucosa via colonoscopy as described by Roper et al.^43^. Tumour formation was confirmed via colonoscope before tissue sampling. Animals heterozygous for the *Apc*^*Min*^ allele of both sexes at the age of 12–16 weeks were used. Samples from *Apc*^*Min/+*^ mice were collected for IF at the onset of clinical signs of intestinal adenomas. For tissue metabolomics (MSI or LC–MS), intestines were flushed with ice-cold PBS, and tissues of interest were dissected and snap frozen.

To study the effect of AHCY inhibition in vivo, Villin-Cre^ERT2^ *Apc*^*fl/fl*^ (APC) and Villin-Cre^ERT2^ *Apc*^*fl/fl*^ *Kras*^*G12D/+*^ (APC KRAS) mice were treated daily with DZNeP.HCl (5 mg per kg body weight i.p.; Carbosynth) or vehicle (PBS) from day 1 after i.p. tamoxifen administration (Extended Data Fig. 8a,b). Animals were injected with BrdU (i.p.) 2 h before sampling tissues. Tissues were collected on day 4 (APC) or day 3 (APC KRAS). To test the effects of long-term DZNeP treatment, WT C57/BL6 mice were treated from 50 (±2) days of age with vehicle (PBS; *n* = 10) or DZNeP.HCl (2 mg per kg body weight i.p.; *n* = 10) using weekly cycles of 4 d of daily treatment, followed by 3 d of no treatment. Body weights were recorded three times per week and mice were euthanized at 93 (±2) days of age. *Apc*^*Min/+*^ mice were treated from day 50 (±2) with vehicle (PBS; *n* = 11) or DZNeP.HCl (2 mg per kg body weight i.p.; *n* = 12) using weekly cycles of 4 d of daily treatment (that is, regime 1), or with DZNeP.HCl (5 mg per kg body weight i.p.; *n* = 10) twice per week (that is, regime 2). Tissues were collected at 85 (±2) days of age and macroscopic intestinal tumour burden was scored. Blood analysis was performed using a ProCyte Dx Hematology Analyzer (IDEXX).

To study tumour-specific distribution of glycocholic acid (GA; Extended Data Fig. 3), ^13^C-GA (CLM-191, CK Isotopes) was administered by oral gavage to tumour-bearing animals (APC, spatially localized Cre induction) at 75 mg per kg body weight (vehicle: 10% dimethylsulfoxide, 0.5% hydroxypropyl methylcellulose + 0.1% Tween-80). Tissues were harvested and snap frozen for downstream analysis 8.5 h after administration.

### Haematoxylin & eosin, immunohistochemistry and RNA-ISH

Intestinal tissues were fixed in 10% neutral buffered formalin or methacarn (methanol:chloroform:acetic acid at a 4:2:1 ratio). All H&E, IHC and ISH staining was performed on 4-µm formalin-fixed paraffin-embedded sections (FFPE) that had previously been heated at 60 °C for 2 h.

Standard protocols were used for H&E staining. FFPE sections for BrdU (347580, Becton Dickinson) IHC staining were loaded into an Agilent pretreatment module to be dewaxed and undergo heat-induced epitope retrieval using high pH target retrieval solution (TRS; K8004, Agilent). The sections were heated to 97 °C for 20 min in high pH TRIS buffer. After heat-induced epitope retrieval, the sections were rinsed in flex wash buffer (K8007, Agilent) before being loaded onto the Agilent autostainer. Mouse on Mouse blocking reagent (MKB-2213, Vector Labs) was applied to the sections for 20 min before washing with wash buffer. The sections underwent peroxidase blocking (S2023, Agilent) for 5 min and were then rinsed with flex buffer before applying BrdU antibody to the sections at a previously optimized dilution (1:250) for 35 min. The sections were washed with flex wash buffer before application of mouse envision secondary antibody (K4001, Agilent, undiluted) for 30 min. Sections were rinsed with flex wash buffer before applying Liquid DAB (K3468, Agilent) for 10 min. The sections were washed in water and counterstained with haematoxylin z (RBA-4201-00A, CellPath).

ISH for *Lgr5* (312178) and *Olfm4* (311838; both from Advanced Cell Diagnostics) mRNA was performed using RNAScope 2.5 LSx (brown) detection kit (322700, Advanced Cell Diagnostics). BaseScope ISH detection for *Ahcy* (1174218) mRNA was performed using BaseScope LS (red) reagent kit (323600; Advanced Cell Diagnostics). ISH staining was performed on a Leica BOND Rx autostainer strictly according to the manufacturer’s instructions^44^. Images were analysed using HALO software (Indica Labs). To improve visual interpretation, images for *Lgr5* were processed using ImageJ v2.9.0/1.53t to show ISH staining in red.

### Immunofluorescence

#### Intestines from wild-type and *Apc*^Min/+^ mice and organoids

Intact intestinal tissues were flushed with PBS followed by 10% buffered formalin (Sigma-Aldrich). Intestines were then fixed as a Swiss roll in 10% buffered formalin overnight with gentle rocking. The tissues were rinsed in 70% ethanol before embedding in paraffin wax. Four-micron sections were dewaxed and rehydrated by two-time immersion in xylene for 10 min, then two-time immersion in 100% ethanol for 10 min, followed by subsequent immersion in 95, 70, 50 and 25% ethanol for 5 min, and rinsed in water. Antigen retrieval was performed for 20 min at high temperature with 10 mM citrate, pH 6.0. Slides were washed with PBS and blocked with 1% BSA and 0.1% Triton X-100 in PBS.

Organoids were seeded in eight-well slide chambers (Thermo Scientific Nunc, 154526) and fixed in prewarmed 4% formalin for 25 min and then rinsed and permeabilized by incubating for 1 h in 1% BSA and 0.1% Triton X-100 in PBS.

Tissue or cell culture slides were incubated with an appropriate dilution of primary antibody (rabbit anti-AHCY (ProteinTech, 10757-2-AP; 1:100 dilution) and mouse anti-5m-cystosine (Abcam, Ab10805; 1:100 dilution)) in a humidity chamber overnight at 4 °C. Slides were washed five times in PBS for 5 min with gentle rocking, followed by a 2-h incubation with a 1:250 dilution of either anti-rabbit conjugated to Alexa Fluor 555 or anti-mouse conjugated to Alexa Fluor 488, as appropriate, in blocking solution for 1 h, and washed again five times in PBS for 5 min with gentle rocking. Slides were mounted to a coverslip with Vectashield (Vector Laboratories) containing 0.1 µg ml^−1^ DAPI. Images were captured with a Leica SP5 confocal microscope.

#### Intestines from Villin-Cre^ERT2^*Apc*^fl/fl^ (*Kras*^G12D/+^) mice and human samples

IF was performed on 4-µm FFPE sections that had previously been kept at 60 °C for 2 h. FFPE sections for AHCY (10757-2-AP, Proteintech) IF staining were loaded onto a Leica Bond Rx autostainer. FFPE sections underwent on-board dewaxing (AR9222, Leica) and antigen retrieval using ER1 solution (AR9661, Leica) for 40 min at 95 °C. Sections were rinsed with Leica wash buffer (AR9590, Leica) before a 10% normal goat serum (X090710, Agilent) solution was applied for 30 min. Sections were rinsed with wash buffer before AHCY antibody was applied at a 1:500 dilution. Sections were rinsed with wash buffer and anti-rabbit IgG 647 diluted at a 1:250 ratio applied for 30 min before rinsing with wash buffer. DAPI was applied to the sections before rinsing with wash buffer and then the sections were coverslipped using pro-long gold (P10144, Thermo Fisher). Images were captured using the Keyence BZ-X810 microscope.

### RNA sequencing

For RNA-seq of tissues from WT, APC or APC KRAS mice, 1 µg of RNA was prepared in 50 µl. RNA-seq was performed using an Illumina TruSeq RNA sample prep kit, then run on an Illumina NextSeq using the High Output 75 cycles kit (2 × 36 cycles, paired-end reads, single index). For RNA-seq of tissues from APC mice treated with vehicle or with DZNeP, 1 µg of RNA was prepared in 25 µl. RNA-seq was performed using an Illumina Stranded mRNA Prep kit, then run on an Illumina NextSeq 500 using the High Output 75 cycles kit (2 × 36 cycles, paired-end reads, dual indexed).

The raw sequence quality was assessed using the FastQC algorithm version 0.11.8. Sequences were trimmed to remove adaptor sequences and low-quality base calls, defined by a Phred score of <20, using the Trim Galore tool version 0.6.4. The trimmed sequences were aligned to the mouse genome build GRCm38.98 using HISAT2 version 2.1.0, then raw counts per gene were determined using FeatureCounts version 1.6.4. Differential expression analysis was performed using the R package DESeq2 version 1.22.2, and PCA was performed using R base functions.

### Single-cell RNA sequencing

Villin-Cre^ERT2^ *Apc*^*fl/fl*^ (APC; *n* = 2) mice received two i.p. injections of 2 mg tamoxifen and were sampled 4 d after induction. Segments from the small intestine (5–15 cm) and colon (mid colon) were collected and processed immediately for scRNA-seq. Briefly, samples were homogenized and digested using a Mcllwain Tissue Chopper and the GentleMACS Octo Dissociator (Miltenyi Biotec, 130-096-427) in combination with the mouse tumour dissociation kit (Miltenyi Biotec, 130-096-730). Debris was then removed using the debris removal solution kit (Miltenyi Biotec, 130-109-398). Cells were sorted using a FACSAria sorter (BD Biosciences) and DAPI (Invitrogen, D1306) to remove dead cells. A total of 40,000 cells were loaded onto each channel of Chromium Chip G using reagents from the 10x Chromium Single-Cell 3′ v3 Gel Bead Kit and Library (10x Genomics) according to the manufacturer’s protocol. The libraries were analysed using the Bioanalyzer High Sensitivity DNA Kit (Agilent Technologies). scRNA-seq libraries were sequenced on the Illumina NovaSeq 6000 with paired-end 150-bp reads. Sequence alignment of single-cell data to the mm10 genome was performed using the count tool from the Cell Ranger package version 6.1.2 according to the developer’s instructions, generating barcodes, features and matrix output files for each sample. Subsequent analysis was done using R version 4.1.1 using Seurat version 4.0.4. Samples were input using the Read10X function, filtering to include cells with a minimum of 100 expressed genes and genes that were present in at least three cells, then further filtered to only include cells with <5% mitochondrial genes, <10% haemoglobin genes, >100 genes per cell and >400 reads per cell. Samples were then integrated by RPCA using the IntegrateData function before being scaled and normalized. Dimension reduction was then performed using PCA before clustering was performed using the FindNeighbours and FindClusters functions. Marker genes for individual clusters were determined using the FindAllMarkers function. Cell types were annotated using CellTypist and custom gene lists.

### Organoid studies

Intestines from WT C57BL/6J mice were sliced longitudinally, rinsed in calcium-free and magnesium-free PBS (PBS-CMF) with vigorous shaking, and cut into 2-cm segments. Villi were scraped and rinsed away. The tissue was minced and collected in a Falcon tube. The tissues were washed approximately ten times by resuspension in fresh PBS-CMF, pelleting by gravity, aspiration and resuspension in fresh PBS-CMF. The tissue was centrifuged at 200*g* for 5 min. PBS-CMF was aspirated. Crypts were removed by suspension in 10 ml 2 mM EDTA and gentle agitation for 30 min followed addition of 10 ml PBS-CMF and vigorous shaking. EDTA was neutralized by 5 ml PBS with calcium and magnesium. The crypt-free tissue was allowed to settle to the bottom of the tube and the crypt-containing supernatant was collected. Crypts were pelleted and washed 2× with Advanced DMEM-F12 by centrifugation at 200*g* for 5 min. The crypts were resuspended in Matrigel and seeded as drops onto culture dishes. After the Matrigel solidified, a 1:1 dilution of Advanced DMEM-F12 medium with 10 mM HEPES, 1× B27, 1 mM *N*-acetylcysteine, 50 ng ml^−1^ EGF, 500 ng ml^−1^ R-spondin and 200 ng ml^−1^ Noggin WNT conditional medium^45^ was added. After the crypts formed spheres, the WNT conditional medium was removed, which allowed the spheres to differentiate and bud.

APC^Min^ adenomas were isolated by slicing the adenoma-bearing tissues longitudinally, rinsing with PBS-CMF, and excising adenomas with forceps. Adenomas were dissociated by incubating with 1 mg ml^−1^ collagen dispase in Advanced DMEM-F12 for 1 h. Dispase was neutralized with 5% FBS. Single cells were isolated by filtering and pelleting by centrifugation at 200*g* for 5 min. The cells were resuspended in Matrigel and seeded as drops onto culture dishes. APC-mutant cells were selected by not including WNT or R-spondin in the medium. After the establishment of organoids, organoids were cultured in Advanced DMEM-F12 medium with 10 mM HEPES, 1× B27, 1 mM *N*-acetylcysteine, 50 ng ml^−1^ EGF, 500 ng ml^−1^ R-spondin and 200 ng ml^−1^ Noggin.

Organoids were isolated from the small intestines of Villin-*CreER Apc*^*fl/fl*^ mice as described previously^46^. Organoids were resuspended in Matrigel (BD Biosciences), plated in 24-well plates and supplemented with Advanced DMEM/F12 supplemented with 10 mM HEPES, 2 mM glutamine, N2, B27 (all from Gibco, Life Technologies), 100 ng ml^−1^ Noggin and 50 ng ml^−1^ EGF (both from PeproTech).

To evaluate the effect of AHCY inhibition on growth, organoids were seeded as fragments in Matrigel and cultured in the presence/absence of DZNeP.HCl (0 or 1 μM) for 72 h. Culture medium was removed and organoids were stained for 90 min with SYTO 60 Nucleic Acid Stain (0.5 μM; Thermo Fisher). Fluorescence signal was quantified on a LICOR imaging system.

To study the metabolic effects of AHCY inhibition, organoids were seeded as fragments in Matrigel, supplemented with Advanced DMEM/F12 (containing 10 mM HEPES, 2 mM glutamine, N2, B27, 100 ng ml^−1^ Noggin and 50 ng ml^−1^ EGF) and allowed to proliferate for 48 h. Next, organoids were pretreated for 2 h with DZNeP.HCl (0 or 1 μM final concentration), after which medium was replaced with fresh medium supplemented with ^13^C_5_-methionine (116 μM) and DZNeP.HCl (0 or 1 μM final concentration). After 18 h, cells were washed three times with ice-cold PBS and extracted in 400 μl extraction solution (methanol:acetonitrile:water; 50:30:20 ratio). Samples were centrifuged and analysed by LC–MS on a ZIC-pHILIC column as described below. Extracted organoids were stained with SYTO 60 Nucleic Acid Stain as described above and fluorescence signal was used for data normalization.

To evaluate the effect of AHCY inhibition on protein synthesis, APC organoids were seeded as fragments in 30 µl Matrigel supplemented with Advanced DMEM/F12 (containing 10 mM HEPES, 2 mM glutamine, N2, B27, 100 ng ml^−1^ Noggin and 50 ng ml^−1^ EGF) in the presence/absence of DZNeP.HCl (1 μM). After 72 h, medium was replaced with fresh medium (±DZNeP) and, 6 h later, ^35^S-methionine (PerkinElmer, NEG772002MC) was added at 30 μCi ml^−1^ for 30 min. The organoids were then collected and lysed (buffer: 10 mM Tris pH 7.5, 50 mM NaCl, 0.5% NP40, 0.5% deoxycholate, 0.5% SDS, 10 mM Iodoacetamide). Trichloroacetic acid (12.5% wt/vol) was used for protein precipitation onto glass microfibre paper (Whatman, 1827-024) using a vacuum manifold and this was washed with 70% ethanol and acetone. Scintillation was read on a Wallac MicroBeta TriLux 1450 counter using Ecoscint (SLS Ltd LS271) and normalized to total protein content determined by the BCA assay. Protein synthesis rate was shown as counts per minute over protein content (CPM/μg protein).

For genetic silencing of *Ahcy* in *Apc*-deficient organoids, cells (2e5) were incubated for 4 h as organoid fragments in the presence of lentiviral particles (3e5) in 250 µl Advanced DMEM/F12 (containing 10 mM HEPES, 2 mM glutamine and 8 µg ml^−1^ polybrene). The following lentiviral particles, purchased from Horizon Discovery, were used: VSC6570 (shNTC), V3IMMMCG_11220560 (sh*Ahcy#1*), V3IMMMCG_16165841 (sh*Ahcy#2*) and V3IMMMCG_16576790 (sh*Ahcy#3*). After 4 h of incubation, organoids were plated in Matrigel drops and supplemented with Advanced DMEM/F12 containing 10 mM HEPES, 2 mM glutamine, N2, B27, 100 ng ml^−1^ Noggin, 50 ng ml^−1^ EFG and 10 µM Y-27632 (Cambridge Bioscience). After 24 h, puromycin (3 µg ml^−1^) was added for selection. Organoids lines were expanded, exposed for 24 h to doxycycline 0.5 µg ml^−1^, sorted for GFP positivity using a BD FACSAria, and maintained in medium without doxycycline. To evaluate knockdown efficiency, organoids were seeded as single cells (5,000 cells in 10 µl Matrigel) in Advanced DMEM/F12 (containing 10 mM HEPES, 2 mM glutamine, N2, B27, 100 ng ml^−1^ Noggin and 50 ng ml^−1^ EGF) in the presence/absence of doxycycline (0.5 µg ml^−1^; *n* = 6 wells per condition). After 4 d, medium was replaced with fresh medium supplemented with ^13^C_5_-methionine (116 μM). After 24 h, metabolites were extracted for LC–MS as described above. To evaluate cell growth, organoids were seeded as single cells (2,000 cells in 10 µl Matrigel) in Advanced DMEM/F12 (containing 10 mM HEPES, 2 mM glutamine, N2, B27, 100 ng ml^−1^ Noggin and 50 ng ml^−1^ EFG) in the presence/absence of doxycycline (0.5 µg ml^−1^). Medium was changed regularly and, after 5 or 6 d, metabolites were extracted for LC–MS and organoids were stained with SYTO 60 Nucleic Acid Stain to evaluate growth as described above.

### High-performance liquid chromatography–mass spectrometry

Frozen tissue fragments were weighed and homogenized in ice-cold extraction solution (20 mg ml^−1^, kept constant between samples for normalization) using ceramic beads and a Precellys Homogenizer (Bertin Instruments). Samples were centrifuged (10 min, 16,000*g*) and the supernatant was analysed as described below.

A Thermo Ultimate 3000 high-performance liquid chromatography (HPLC) system was equipped with a ZIC-pHILIC column (SeQuant; 150 mm by 2.1 mm, 5 μm; Merck KGaA), with a ZIC-pHILIC guard column (SeQuant; 20 mm by 2.1 mm) for metabolite separation. Cell or tissue extracts were injected (5 μl) and metabolite separation was obtained as described before^47^. The HPLC system was coupled with a Q Exactive Plus Orbitrap Mass Spectrometer (Thermo Fisher Scientific) used with a resolution of 70,000 at 200 mass-to-charge ratio (*m/z*), electrospray ionization and polarity switching mode across a mass range of 75 to 1,000 *m/z*. Mass accuracy was below 5 ppm. Untargeted metabolomics was performed as previously described^47^. In brief, where required for metabolite identification, a mixture of all samples within an experiment was analysed in both positive and negative single ionization mode using data-dependent fragmentation (ddMS2). Data was acquired using Xcalibur software (v4.3, Thermo Scientific). Untargeted data analysis was performed using Compound Discoverer (v3.2, Thermo Scientific). Retention times were aligned across all sample data files (maximum shift 2 min, mass tolerance 5 ppm). Unknown compound detection (minimum peak intensity 5e5) and grouping of compound adducts were carried out across all samples (mass tolerance 5 ppm, retention time (RT) tolerance 0.7 min). Missing values were filled using the software’s Fill Gap feature (mass tolerance 5 ppm, S/N tolerance 1.5). Metabolite identification was achieved by matching the mass and RT of observed peaks to an in-house database generated using metabolite standards (mass tolerance 5 ppm, RT tolerance 0.5 min), Peak annotations were confirmed using mzCloud (ddMS2) database search (precursor and fragment mass tolerance of 10 ppm, match factor threshold 50) and searching predicted compositions (mass tolerance 5 ppm, minimum spectral fit and pattern coverage of 30% and 90%, respectively) against the HMDB database. Targeted data analysis was performed using Tracefinder (v4.1, Thermo Scientific). Statistical tests were performed using Compound Discoverer, Perseus (1.6.2.2)^48^ and GraphPad Prism 9.

#### Detection of ^12^C-glycocholic acid and ^13^C-glycocholic acid

Single reaction monitoring mode was used to detect glycocholic acid on a Altis QQQ Mass Spectrometer equipped with a Vanquish LC system (Thermo Fisher Scientific). Separation of metabolites was performed on a Acquity HSS T3 column (Waters; 150 mm by 2.1 mm, 1.8 μm). The mobile phase consisted of solvent A (water with 0.1% formic acid) and solvent B (acetonitrile with 0.1% formic acid) using the following gradient: 0 min 20% B, 8 min 95% B and 10 min 20% B, at a constant flow rate of 0.3 ml min^−1^. The injection volume was 5 µl. Two transitions were optimized using an authentic standard of glycocholic acid (glycine-1-^13^C, CLM-191-PK, Cambridge Isotope Laboratories), from the negative precursor ion (*m/z* 465) to product ions (*m/z* 402 and *m/z* 75). Total cycle time was 0.8 s and Q1 resolution (FWHM) was 0.7 and Q3 resolution (FWHM) was 1.2. For each transition, the collision energy applied was optimized to generate the greatest possible signal intensity. The optimized source parameters were: spray voltage, 2,500 V; sheath gas, 35; Aux gas, 7; ion transfer tube temperature, 325 °C; vaporizer temperature, 275 °C; and RF lens, 105. Data acquisition was performed using Xcalibur 4.1 software.

### Rapid evaporative ionizing mass spectrometry

REIMS was performed on intestinal epithelium extracts, prepared as previously described^49^. An Erbe VIO 50C electrosurgical generator (Erbe Elektonedizin) operated in bipolar mode at 25 W was used to power the sampling forceps. Samples were allowed to reach room temperature before sampling, a portion of each pellet was removed using one tip of the forceps, the two electrodes of the forceps were brought into close proximity and the forceps were activated using a foot switch. The generated smoke was aspirated and directed to the REIMS interface using a 2-m-long Tygon tube. An isocratic solvent manager (Waters) was used to introduce propan-2-ol to the Venturi of the REIMS interface at a flow rate of 0.1 ml min^−1^, where it was mixed with the aspirated aerosol. REIMS data were acquired in negative ion mode (50–1,500 *m/z*) using a Waters Xevo G2-XS QToF mass spectrometer fitted with an REIMS interface (30,000 mass resolving power at *m/z* 956, <1 ppm RMS mass accuracy; Waters).

Data were converted from proprietary.RAW format to imzML using ProteoWizard^50^ and imzML Converter^51^ and analysed using SpectralAnalysis^52^ in MATLAB (2019b). Data were pre-processed using interpolation rebinning using a bin width of 0.001 Da, to create a consistent *m/z* axis to generate a mean spectrum. The mean spectrum was then peak picked using a gradient method, and the top 2,000 peaks selected for further processing. The regions where the forceps were active were then identified by clustering the data using *k*-means clustering using the cosine distance with *k* = 2. Data were then l_2_ normalized before further analysis by *t*-SNE and clustering.

*t*-SNE was applied to these data using the MATLAB function ‘tsne’ (MATLAB v2017a, Statistics and Machine Learning Toolbox) reducing the data to three dimensions using the cosine distance metric and the default hyperparameters^53^. Clustering was then performed on the *t*-SNE reduced data using the MATLAB ‘kmeans’ function with the Euclidean distance and ten replicates and five clusters (five clusters were chosen as there were five genotypes included in the study).

Following the segmentation, pairwise mean intensity log_2_ fold change and two-sided *t*-test *P* values were calculated between the data for each *m/*z from each genotype. The discriminating ions from this analysis (absolute fold change > 1.5, *P* value < 0.05, and significant after Benjamini–Hochberg FDR correction (*q* = 0.05) were then matched to the human metabolome database^54^ using custom MATLAB scripts^55^. For each tentative molecule that was identified, the biochemical pathways in which that molecule was involved were then identified. Pathways were then reported in descending order of the number of unique discriminating *m/z* that contained a molecule for that pathway (Supplementary Table 1). Pathways from the same HMDB subclass were curated to provide a more concise list of pathways to review (for example, Phosphatidylcholine Biosynthesis PC(22:1(13Z)/20:0), and Phosphatidylcholine Biosynthesis PC(22:0/24:0). No MS/MS was performed on these data. Tentatively identified molecules belonging to pathways shown in Supplementary Table 1 are described in Supplementary Table 2. However, additional isomeric and isobaric molecules may also be assigned to these *m/z*. For both Supplementary Tables 1 and 2, ambiguity for lipid annotations has been removed due to inability to differentiate side-chain and double-bond positions (for example, Phosphatidylcholine Biosynthesis PC(22:1(13Z)/20:0) is shortened to Phosphatidylcholine Biosynthesis PC(42:1)). Relevant scripts are available at https://github.com/oycxyd/JVVNatMet/tree/main/Cell%20pellets/Cell%20pellet%20REIMS%20scripts.

### Mass spectrometry imaging

Frozen tissue samples were prepared for MSI as described previously^56^.

#### DESI-MSI

DESI-MSI was carried out using a Xevo G2-XS QToF instrument (Waters) and a developmental sprayer incorporating a recessed capillary (Waters). Solvent comprising 95:5 methanol:water (Optima grade, Fisher Scientific) was used. The DESI sprayer was operated using the following parameters: 0.8 kV, 4 bar Nitrogen gas pressure, 50 V cone, 100 °C ion block temperature, room temperature inlet capillary, sprayer angle of 80°, 2 mm distance to the inlet capillary, 2 mm distance from sprayer nozzle to sample. Before data acquisition DESI signal intensity was optimized using black permanent marker to reach ion intensity for *m/z* 666 of >1e^6^ and on bovine brain homogenate tissue of >1e^4^ for basepeak in lipid *m/z* region (700–900). Mass calibration was carried out daily or before each sample analysed, whichever was more frequent, for a *m/*z range of 50–1,200 using mass spectra derived from a polylactic acid-coated glass slide. Data were also acquired with this mass range for pixel size 75 × 75 µm at a scan rate of two pixels per second. DESI MS/MS was undertaken using the same conditions and instrument. The instrument was operated in the MS/MS mode with RF voltage set to 7.4. For identification of the precursor ion that was assigned as glucose (Extended Data Fig. 2), the collision energy used was 10.

Volcano plot produced from MSI data as shown in Extended Data Fig. 4a was produced from tumour subregions of MSI data defined by *t*-SNE and *k*-means clustered regions of interest, which were observed to correlate with tumour regions in the corresponding H&E-stained tissue sections from the same tissues. Pixel data were RMS normalized, zeros were removed, and two-sided *t*-tests were performed in Python 3.7.9 (ttest_ind, scipy.stats package). Fold changes were calculated from average intensity values and plotted against *P* values, where 1.5 and 0.05, respectively, and significant after Benjamini–Hochberg FDR correction (q = 0.1), were considered as thresholds in the displayed figures. Python script is available at https://github.com/oycxyd/JVVNatMet/tree/main/DESI%20volcano.

#### MALDI-MSI

MALDI-MSI was carried out using a Synapt G2-Si QToF instrument (Waters) fitted with a uMALDI ion source^57^. The samples were coated with 9-AA (Merck Life Science) at 10 mg ml^−1^ in 80:20 ethanol:water by TM Sprayer (HTX Technologies) with a temperature of 65 °C, flow rate of 0.06 ml min^−1^, nozzle velocity of 1,200 mm s^−1^, four passes, 3 ml track spacing and CC pattern alternation. The instrument was calibrated with Red Phosphorus to an RMS mass accuracy of <1 ppm, and the instrument method was set as follows: pixel size of 45 × 45 µm, sensitivity mode, negative ion mode, *m/z* range of 50–1,200, laser repetition rate of 2,500 Hz and scan time of 0.5 s.

DESI and MALDI-MSI data obtained from APC and APC KRAS tumours were also comprehensively evaluated for potential ion signal corresponding to SAM, SAH, homocysteine, cystathionine and methionine. On-tissue ion signal of sufficient signal-to-noise or accurate mass error was not identified for these ions. Metabolite standards were readily detected by DESI (Supplementary Table 7) but the lack of detection of endogenous ions in tissues derived from genetically engineered mouse models suggests these are below the limit of detection for the systems used in this study.

#### DESI and MALDI-MSI *t*-SNE data analysis

Data were converted from proprietary.RAW format to imzML using ProteoWizard^50^ and imzML Converter;^51^ and analysed using SpectralAnalysis^52^ and in-house-developed scripts in MATLAB (2017a). MSI data were not normalized and were used raw after conversion to imzML. The top 4,000 peaks of each dataset were used to create a datacube and perform spatial segmentation by *k*-means clustering, with the similarity metric set to ‘cosine’ and the number of clusters set to 4. Tissue and background were manually labelled by comparing the optical images with the spatial segmentation results. The total spectrum for the tissue was created by summing all pixels belonging to the clusters categorized as such. Following removal of background pixels, *t*-SNE was performed on the tissue-only pixels using the MATLAB function ‘tsne’, the data were reduced to three dimensions, and the spectral similarity metric used was correlation^58^. The remaining *t*-SNE parameters (for example, perplexity) were set to their MATLAB default values. The *k*-means cluster value (*k*) was determined using the elbow method^59^ unless otherwise stated. Data representation shown in Fig. 2e was produced from background-subtracted data with subsequent *k* = 3 *k*-means clustering applied.

#### LESA MS/MS

LESA CID MS/MS was carried out using a SYNAPT G2-Si QToF (Waters) and the LESA Advion TriVersa NanoMate was used as the ionization source^60^. A surface extraction solvent of 95:5 methanol:water was used for all experiments. LESA parameters used for analysis were: solvent volume 4 µl, solvent depth 1 mm up from the bottom of the reservoir, dispensed 2 µl, delayed 2 s after dispensing, aspirated 3.5 µl, repeated mix two times, delayed 2 s after aspirate. Negative ion nano-ESI was used for the MS/MS analysis with spraying parameters of 0.3 psi gas pressure and 1.4 kV for applied voltage. The SYNAPT G2-Si was operated in negative ion MS/MS mode for precursor ion at *m/z* 464. A range of collision energies were used with the data averaged.

### Clinical studies

#### Metabolic profiling of clinical samples by REIMS

Colorectal samples were collected from individuals undergoing colorectal resection surgery with carcinoma, adenoma and normal tissue sampled. All adults undergoing endoscopic or surgical resection were eligible for inclusion in the study. Individuals with irritable bowel disease and hereditary polyposis were excluded from the study. Samples were collected under a subcollection of Imperial College Healthcare Tissue Bank (ref. 17/WA/0161 under HTA license 12275) and written and informed consent was collected pre-operatively. Following resection, samples were transported fresh to the histopathology department where they were cut and sampled for research under supervision of a consultant histopathologist. After this, samples were stored at −80 °C until sampled for MS or extracted for genomic analysis. The average age of the participants was 69 years. While a balanced ratio of male to female participants was desired, this was not always possible due to the ability to sample based on size and the availability of tissue for research. Also, some samples did not contain enough DNA on extraction for full exome sequencing and therefore all viable samples were used, resulting in a ratio of male (*n* = 10) and female (*n* = 14). Sex- and gender-dependent analysis was not performed. Participation in this research was not incentivized and inclusion was based on tissue bank samples from procedures taking place as part of routine clinical care.

Ex vivo analysis was carried out by cutting the tissues with a monopolar diathermy handpiece at 25 W. The surgical aerosol was then transferred to the REIMS source via PTFE tubing. REIMS analysis was carried out on a Xevo G2-XS ToF mass spectrometer (Waters) fitted with an REIMS source. Data were generally collected in negative ionization, continuous mode over the *m/z* range of 50–1,200. Isopropanol was co-aspirated with the surgical aerosol to provide a matrix to increase sensitivity at a rate of 0.1 µl min^−1^. After MS analysis, samples were placed into formalin and FFPE blocks, and histologically verified by slicing to 10-µm sections and capturing onto a glass slide. The slides were then H&E stained following the standard National Health Service (NHS) protocol and examined by a consultant histopathologist for verification of exact tissue type. Sections of samples analysed by REIMS were extracted using a QIAamp DNA Micro Kit (Qiagen) for determination of their driver mutation status. DNA quantification was carried out using the Qubit assay and whole-exome sequencing performed by Genewiz (Azenta Life Sciences).

Data were pre-processed using an in-house pipeline in MATLAB v2020a. The predictive performance of the models built by machine learning algorithm was evaluated by computing standard metrics, including the true positive rate, true negative rate, false positive rate (1 − true negative rate) and balanced accuracy. To further enhance the model performance and robustness, optimization by means of feature refinement^61^ was carried out to remove metabolic features that were more affected by spurious background factors (for example, instrumental conditions), and keeping only the significant contributors to the classification task. *t*-SNE was also applied to the feature refined data (containing only 50 features) for visualization. A three-dimensional projection was achieved with the following hyperparameters that have been empirically fine-tuned for the clinical data: initialization method = ‘pca’, perplexity = 5, learning_rate = 50. All other hyperparameters were kept default. Relevant codes are available at https://github.com/oycxyd/JVVNatMet/tree/main/iKnife.

#### AHCY gene/protein expression in a colorectal cancer adult cohort

A retrospective cohort of 787 adults with stage I–III CRC was utilized to determine any prognostic value of *AHCY* gene expression. Participants were staged using the 5th edition of TNM staging and underwent surgical resection with curative intent between 1997 and 2013 within Greater Glasgow and Clyde NHS board. Participants who received neoadjuvant therapy or died within 30 d of surgery were excluded from analysis, which left 701 adults included in analysis. The median age of participants was 71 years, ranging from 21 to 98 years. Information regarding sex was collected from the clinical portal and the cohort consisted of 45% female and 55% male adults. Data were deposited with Glasgow Safehaven (GSH21ON009). This study was approved by the Research Ethics Committee of the West Glasgow University Hospitals NHS Trust (NHS GG&C REC ref. 22/WS/0020), in accordance with Human Tissue (Scotland) Act 2006, which included policy on consent. H&E-stained full tumour resections were annotated by two observers (P.H. and K.P.) to select epithelium-rich regions for sequencing. Whole-transcriptome profiling was performed on tissue extracted from these regions using TempOSeq technology as previously described^62^. Raw gene counts were normalized using DESeq2 package in R studio version 1.4 (RStudio). Continuous gene counts were segregated into high and low groups using an optimal cut-off point determined using the survminer package in R studio based on cancer-specific survival. Kaplan–Meier survival analysis was utilized to determine the association between *AHCY* expression and cancer-specific survival in SPSS version 25 (IBM). A subset of samples from the same patient cohort were used to analyse AHCY protein expression using IF as described above. Normal human colon (BB060067) was obtained from the Queen Elizabeth University Biorepository by agreement.

### Bioinformatic analysis

Bioinformatic analyses were performed using public data portals and R (v4.1.1). To study the gene expression of *AHCY* across the different CMS classes of CRC (Extended Data Fig. 5b), individuals with colonic adenocarcinoma from the TCGA database were assigned to CMS classes using the CMSclassifier R package as described in Guinney et al.^19^. Differential gene expression for each CMS group relative to all others was then performed using the R package limma (version 3.48.3) as follows. Linear models were fitted per gene using the lmFit() function, then statistics were calculated using the eBayes() function before being extracted and multiple-testing correction performed using the topTable() function, all with default settings.

### Data visualization and statistical analysis

Unless mentioned differently, data were plotted and analysed using Microsoft Excel (2008 and 2016) and GraphPad Prism 9.0.

### Reporting summary

Further information on research design is available in the Nature Portfolio Reporting Summary linked to this article.

## Supplementary information

Reporting Summary

Supplementary Tables 1–7 Supplementary Table 1: Pathway analysis of ions detected by REIMS (*m/z* 600–1,500; negative polarity), and found to discriminate between genotypes in epithelial extracts of WT, KRAS, APC, APC KRAS and APC KRAS PTEN mice. Supplementary Table 2: Tentative identification of ions detected by REIMS (*m/z* 600–1,500; negative polarity) and found to discriminate between genotypes in epithelial extracts of WT, KRAS, APC, APC KRAS and APC KRAS PTEN mice. *P*values (obtained from two-tailed *t*-tests) in bold are significant after Benjamini–Hochberg FDR correction (*q* = 0.05). Supplementary Table 3: List of ions (*m/z* values) driving the classification of human colorectal samples analysed by REIMS (negative polarity). Supplementary Table 4: List of ions (*m/z* values) used to construct volcano plot (Extended Data Fig. 4a) showing metabolic differences between tumour epithelial regions of APC and APC KRAS tumours analysed by DESI-MSI (negative polarity). *P* values obtained from two-tailed *t*-tests. Supplementary Table 5: List of molecules (neutral mass) used to construct volcano plot (Extended Data Fig. 4c) showing metabolic differences between bulk tissue extracts from APC and APC KRAS tumours analysed by LC–MS (polarity switching). *P*values obtained from two-tailed *t*-tests. Supplementary Table 6: Summary of sample sizes for in vivo experiments. Supplementary Table 7: DESI analysis of metabolite standards

## Source data

Source Data Fig. 1 Statistical source data.

Source Data Fig. 2 Statistical source data.

Source Data Fig. 3 Statistical source data.

Source Data Fig. 4 Statistical source data.

Source Data Extended Data Fig. 1 Statistical source data.

Source Data Extended Data Fig. 3 Statistical source data.

Source Data Extended Data Fig. 4 Statistical source data.

Source Data Extended Data Fig. 5 Statistical source data.

Source Data Extended Data Fig. 7 Statistical source data.

Source Data Extended Data Fig. 8 Statistical source data.

Source Data Extended Data Fig. 9 Statistical source data.

Source Data Extended Data Fig. 10 Statistical source data.

## Data Availability

The RNA and DNA sequencing data used in this study are publicly available through the Gene Expression Omnibus under accession numbers GSE168478, GSE197316, GSE229639 and GSE229638, and through the Sequence Read Archive under accession numbers PRJNA984203, and PRJNA997336. Mass spectrometry data are available through https://massive.ucsd.edu/ (dataset: MSV000092468). Publicly available databases used in this study are accessible through https://www.cbioportal.org/, https://www.proteinatlas.org/ and https://singlecell.broadinstitute.org/single_cell/. Source data are provided with this paper. All other data are available from the corresponding authors on reasonable request.

## Code availability

Processing scripts used for data mining and presentation are available at https://github.com/oycxyd/JVVNatMet.
