# Supplementary material for: Metabolic profiling stratifies colorectal cancer and reveals adenosylhomocysteinase as a therapeutic target
Source: Nat Metab. 2023 Aug 14;5(8):1303–18. doi: 10.1038/s42255-023-00857-0 (PMC10447251; doi:10.1038/s42255-023-00857-0)
Supplement: Supplementary file 1 — Reporting Summary [file 42255_2023_857_MOESM1_ESM.pdf]

nature portfolio

Corresponding author(s): Owen Sansom
Johan Vande Voorde

Last updated by author(s): Jun 27, 2023

Reporting Summary

Nature Portfolio wishes to improve the reproducibility of the work that we publish. This form provides structure for consistency and transparency in reporting. For further information on Nature Portfolio policies, see our Editorial Policies and the Editorial Policy Checklist.

Statistics

For all statistical analyses, confirm that the following items are present in the figure legend, table legend, main text, or Methods section.

n/a	Confirmed	
☐	☒	The exact sample size (*n*) for each experimental group/condition, given as a discrete number and unit of measurement
☐	☒	A statement on whether measurements were taken from distinct samples or whether the same sample was measured repeatedly
☐	☒	The statistical test(s) used AND whether they are one- or two-sided<br>*Only common tests should be described solely by name; describe more complex techniques in the Methods section.*
☒	☐	A description of all covariates tested
☐	☒	A description of any assumptions or corrections, such as tests of normality and adjustment for multiple comparisons
☐	☒	A full description of the statistical parameters including central tendency (e.g. means) or other basic estimates (e.g. regression coefficient) AND variation (e.g. standard deviation) or associated estimates of uncertainty (e.g. confidence intervals)
☐	☒	For null hypothesis testing, the test statistic (e.g. *F*, *t*, *r*) with confidence intervals, effect sizes, degrees of freedom and *P* value noted<br>*Give P values as exact values whenever suitable.*
☒	☐	For Bayesian analysis, information on the choice of priors and Markov chain Monte Carlo settings
☒	☐	For hierarchical and complex designs, identification of the appropriate level for tests and full reporting of outcomes
☒	☐	Estimates of effect sizes (e.g. Cohen's *d*, Pearson's *r*), indicating how they were calculated

Our web collection on statistics for biologists contains articles on many of the points above.

Software and code

Policy information about availability of computer code

Data collection

Xcalibur software (v4.3, Thermo Scientific)
MassLynx v4.1
HDImaging v1.4
LICOR Image Studio

Data analysis

Microsoft Excel 2016&2008
Graphpad Prism version 9
Compound Discoverer (v3.2, Thermo Scientific)
Tracefinder (v4.1, Thermo Scientific)
LICOR Image Studio Lite
MatLab v2017a/2019b/R2020a
imzMLConverter
ProteoWizard
Python 3.7.9
SpectralAnalysis v1.4.0
HALO V2.0.1145 (Indica Labs)
edgeR v3.28.1
Limma (version 3.48.3)
R v4.1.1
R studio version 1.4

SPSS version 25
Trim Galore version 0.6.4
HISAT2 version 2.1.0
FeatureCounts version 1.6.4
DESeq2 version 1.22.2
imageJ v2.9.0/1.53t

For manuscripts utilizing custom algorithms or software that are central to the research but not yet described in published literature, software must be made available to editors and reviewers. We strongly encourage code deposition in a community repository (e.g. GitHub). See the Nature Portfolio guidelines for submitting code & software for further information.

Data

Policy information about availability of data

All manuscripts must include a data availability statement. This statement should provide the following information, where applicable:

- Accession codes, unique identifiers, or web links for publicly available datasets
- A description of any restrictions on data availability
- For clinical datasets or third party data, please ensure that the statement adheres to our policy

The RNA sequencing data used in this study are publicly available through the Gene Expression Omnibus (GEO) with accession numbers: GSE168478, GSE197316, GSE229639, and GSE229638. DNA sequencing data is available through SRA, accession number PRJNA984203. All other data are available from the corresponding authors on reasonable request. Publicly available databases used in this study are accessible through: https://www.cbioportal.org/; https://www.proteinatlas.org/; https://singlecell.broadinstitute.org/single_cell.

Research involving human participants, their data, or biological material

Policy information about studies with human participants or human data. See also policy information about sex, gender (identity/presentation), and sexual orientation and race, ethnicity and racism.

Reporting on sex and gender

CRC PATIENT COHORT FOR REIMS
Sex was assigned based on medical records. Due to the limited number of samples (n=10 male; n=14 female), and the observational nature of the study, no sex/gender-based analyses were performed.

CRC PATIENT COHORT FOR TEMPOSEQ/IMMUNOFLUORESCENCE
Sex was assigned based on medical records. AHCY expression levels are reported disaggregated for sex in Statistical Source Data.

Reporting on race, ethnicity, or other socially relevant groupings

No socially constructed or socially relevant categorization variables were used in this manuscript.

Population characteristics

CRC PATIENT COHORT FOR REIMS
Samples collected from patients undergoing elective colorectal resection by both open and minimally invasive surgery. Whilst a balanced ratio of male to female patients was desired but this was not always possible due to the observational nature of the study and availability of tissue for research. Moreover some samples did not contain enough DNA on extraction for full exome sequencing and therefore all viable samples were used resulting in a ratio of male (n=10) and female (n=14).
The average age of the patient sample set used for this study was 69 years (range: 35-85).

CRC PATIENT COHORT FOR TEMPOSEQ/IMMUNOFLUORESCENCE
A retrospective cohort of 787 stage I-III CRC patients was utilised to determine any prognostic value of AHCY gene expression. Patients were staged using the 5th edition of TNM staging and underwent surgical resection with curative intent between 1997-2013 within Greater Glasgow and Clyde NHS board. The median age of patients was 71 years, ranging from 21-98 years. Information regarding gender was collected from the clinical portal and the cohort consisted of 45% female and 55% male patients. Data were deposited with Glasgow Safehaven (#GSH21ON009).

Recruitment

CRC PATIENT COHORT FOR REIMS
Colorectal samples were collected from patients undergoing elective colorectal resection surgery for adenocarcinoma or complex adenomas of the colon and rectum at Imperial College NHS trust. All adult patients undergoing endoscopic or surgical resection were eligible for inclusion in the study. Normal tissue was also sampled from these participants as a control specimen. Patients undergoing emergency surgery, those who had undergone previous surgery for bowel cancer, or those with irritable bowel disease, inflammatory bowel disease and hereditary polyposis were excluded from the study. Samples were collected under a sub collection of Imperial College Healthcare Tissue Bank (reference 17/WA/0161 under HTA license 12275) and written and informed consent was collected pre-operatively. Samples were transported fresh to the histopathology department where they were cut and sampled for research under supervision of a consultant histopathologist. They were then stored at -80C until sampled for MS or extracted for genomic analysis.

CRC PATIENT COHORT FOR TEMPOSEQ/IMMUNOFLUORESCENCE
This was a retrospectively collected study. Patients who received neoadjuvant therapy or died within 30 days of surgery were excluded from analysis which left 701 patients included in analysis.

Ethics oversight

CRC PATIENT COHORT FOR REIMS
Use of samples for project was approved by Imperial College Healthcare Trust Tissue Bank (reference 17/WA/0161 under HTA license 12275).

Ethics Committee:	HRA - East of England - Cambridge East Research Ethics Committee
Study Title:	Real time tissue characterisation using mass spectrometry
REC reference:	14/EE/0024

CRC PATIENT COHORT FOR TEMPOSEQ/IMMUNOFLUORESCENCE
This study was approved by the Research Ethics Committee of the West Glasgow University Hospitals NHS Trust (NHS GG&C REC reference: 22/WS/0020), in accordance with Human Tissue (Scotland) Act 2006, this included policy on patient consent.

Note that full information on the approval of the study protocol must also be provided in the manuscript.

Field-specific reporting

Please select the one below that is the best fit for your research. If you are not sure, read the appropriate sections before making your selection.

☒ Life sciences ☐ Behavioural & social sciences ☐ Ecological, evolutionary & environmental sciences

For a reference copy of the document with all sections, see nature.com/documents/nr-reporting-summary-flat.pdf

Life sciences study design

All studies must disclose on these points even when the disclosure is negative.

Sample size

For all in vivo experiments, power analyses were carried out to determine cohort sizes based upon effect size and SD derived from unpublished experiments in similar GA models previously carried out within the lab, and from early pilot studies which were carried out within experimental and control cohorts. Power analyses were carried out using the G* power software package 3.1.9.4 (HHU Dusseldorf), typically defining alpha=0.05 and beta=0.2. Animal studies were also carried out respecting the limited use of animals in line with the 3R system: Replacement, Reduction, Refinement. For all other experiments, sample sizes were not statistically pre-determined, but were based upon results from prior experiments in these and related model systems.
For human studies, experiments were carried out on entire cohorts of historic patient samples, with no prospective sample collection carried out as part of this study. For this reason, it was not possible to carry out predetermined sample size calculations. For in vitro studies, where possible, experiments were carried out with the minimum number of biological replicates required to perform statistical comparison. In all other cases, sample sizes have been reported.

Data exclusions

No data were excluded, unless mentioned otherwise.

Replication

For in vivo experiments, individual animals of control and experimental cohorts are biologically unique -unless mentioned differently, replicate data represents analysis of data/samples from independent replicate animals and is denoted by "n". Unless mentioned differently, all experiments were repeated on at least three independent occasions using the same experimental approach, and all attempts at replication were successful.

For in vitro organoid experiments, one organoid line was used per experiment and numbers of independent replicates, along with technical replicates, are clearly indicated in the manuscript. All attempts at replication were successful. Below is a summary of replications of in vitro experiments:

Organoid growth assay +/- DZNeP: 3 independent experiments (4 or 5 technical replicates each)
Methionine tracing in organoids +/-DZNeP: 2 independent experiments (4 technical replicates each)
Immunofluorescence in organoids: single experiment (4 technical replicates)
Methionine tracing in organoids with shNTC & shAhcy to validate knockdown: single experiment (6 technical replicates each)
Growth assays and accompanying metabolomics in organoids with shNTC & shAhcy: 3 independent experiments (12 technical replicates each)
35S methionine incorporation in organoids: 2 independent experiments (3 technical replicates each)

Randomization

To minimise genetic variability, all experimental and control animals were generated on inbred genetic backgrounds. Where possible, control and experimental animals were co-housed independent of genotype and cohorts were comprised of a balance of both male and female animals. In order to reduce the impact of covariates such as gender or housing, animals were recruited to treatment groups in a partially randomised manner while taking these factors into account.

For in vitro studies, no randomization was performed since experiments happened in a single defined genetic background. For downstream analyses such as mass spectrometry-based metabolomics, samples were randomized prior to analysis to avoid technical differences during analysis to affect outcome.

Blinding

For the preclinical models used in this study, different genotypes are associated with differences in disease progression and severity. For animal welfare reasons, researchers were therefore not blinded to genotype during study and data collection. The investigator(s) were blinded to genotype or treatment during data analysis.

Reporting for specific materials, systems and methods

We require information from authors about some types of materials, experimental systems and methods used in many studies. Here, indicate whether each material, system or method listed is relevant to your study. If you are not sure if a list item applies to your research, read the appropriate section before selecting a response.

Materials & experimental systems

n/a	Involved in the study	
☐	☒	Antibodies
☐	☒	Eukaryotic cell lines
☒	☐	Palaeontology and archaeology
☐	☒	Animals and other organisms
☒	☐	Clinical data
☒	☐	Dual use research of concern
☒	☐	Plants

Methods

n/a	Involved in the study	
☒	☐	ChIP-seq
☒	☐	Flow cytometry
☒	☐	MRI-based neuroimaging

Antibodies

Antibodies used

Antibodies, along with their catalog numbers are included within the manuscript -
BrdU (BD Biosciences, #347580)
β-catenin (BD Biosciences, #610154)
AHCY (ProteinTech 10757-2-AP)
5m-Cystosine (Abcam Ab10805)
Mouse envision secondary antibody (K4001, Agilent)

Validation

Antibodies were used according to manufacturers instructions.

The Anti-BrdU antibody, clone B44, is derived from hybridization of Sp2/0-Ag14 mouse myeloma cells with spleen cells from BALB/c mice immunized with iodouridine-conjugated ovalbumin.Bromodeoxyuridine (BrdU) is a uridine derivative that can be incorporated specifically into DNA in place of thymidine. Anti-BrdU identifies BrdU (but not thymidine) in single-stranded DNA, free BrdU, or BrdU coupled to a protein carrier. The antibody also reacts with iodouridine. Development references available from the supplier (https://www.bdbiosciences.com/en-us/products/reagents/flow-cytometry-reagents/clinical-discovery-research/single-color-antibodies-ruo-gmp/purified-mouse-anti-brdu.347580). Tissues derived from control mice (no BrdU administered) were stained as a negative control.

For B-catenin, staining specificity was confirmed in tissues deficient in beta-catenin expression generated from genetically engineered mice. Development references available from the supplier (https://www.bdbiosciences.com/en-us/products/reagents/microscopy-imaging-reagents/immunofluorescence-reagents/purified-mouse-anti-catenin.610153).

AHCY antibody specificity validated by manufacturer using siRNA and Western blot, IF shown previously in Fu et al. (2018; DOI 10.1016/j.chemosphere.2018.05.048).

5m-cytosine antibody according to manufacturer: Raised against the modified ribonucleoside. Specific for the presence of a methyl group on carbon 5 of the pyrimidine ring. Detects modified base 5-methylcytidine found in DNA of plants and vertebrates. Antibody used in 127 publications (https://www.abcam.com/5-methylcytosine-5-mc-antibody-33d3-ab10805.html?productWallTab=ShowAll)

Eukaryotic cell lines

Policy information about cell lines and Sex and Gender in Research

Cell line source(s)

3D organoid lines from mouse intestinal crypts were derived at the Cancer Research UK Beatson Institute and the Francis Crick Institute

Authentication

Since cell lines were derived in house, no authentication was performed

Mycoplasma contamination

All cell lines are routinely tested for mycoplasma and it is considered that the lines used in the study are mycoplasma free.

Commonly misidentified lines
(See ICLAC register)

No commonly misidentified lines were used in the study

Animals and other research organisms

Policy information about studies involving animals; ARRIVE guidelines recommended for reporting animal research, and Sex and Gender in Research

Laboratory animals

Adult male and female genetically engineered mice of a C57BL/6J background were used in this study. Animals entered study at between 6-12 weeks of age, and only once they had reached a minimum body weight of 20g. Mice were assessed for symptoms of ill health at least 3 times per week, and humanely culled at upon reaching a clinical endpoint in line with UK Home Office regulations. Animals were housed in conventional caging, with environmental enrichment on a 12 hour light-dark cycle in a temperature and humidity controlled environment, with access to food and water ad libitum.

Wild animals

No wild animals were used in the study

Reporting on sex	Where applicable, data is reported disaggregated for sex in the statistical source data files. Sex was not considered in study design, but where overt sex-dependent differences were observed, these were reported.
Field-collected samples	No field-collected samples were used in this study.
Ethics oversight	All animal experiments were performed in accordance with UK Home Office regulations (Project licenses 70/8646 and PP3908577 and P609116C5), and were reviewed and approved by the Animal Welfare and Ethical Review Board (AWERB) of the University of Glasgow and the Francis Crick Institute.

Note that full information on the approval of the study protocol must also be provided in the manuscript.
